# Long non-coding RNA DLX6-AS1 is the key mediator of glomerular podocyte injury and albuminuria in diabetic nephropathy by targeting the miR-346/GSK-3β signaling pathway

**DOI:** 10.1038/s41419-023-05695-2

**Published:** 2023-02-28

**Authors:** Jia Guo, Wen Zheng, Yong Liu, Mengwen Zhou, Yan Shi, Min Lei, Chaojie Zhang, Zhangsuo Liu

**Affiliations:** 1 Nephrology Research Center, the First Affiliated Hospital of Zhengzhou University, Zhengzhou, 450052 P. R. China; 2 Institute of Nephrology, Zhengzhou University, Zhengzhou, 450052 P. R. China; 3 Henan Province Research Center for Kidney Disease, Zhengzhou, 450052 P. R. China; 4 Key Laboratory of Precision Diagnosis and Treatment for Chronic Kidney Disease in Henan Province, Zhengzhou, 450052 P. R. China

**Keywords:** Kidney diseases, Experimental models of disease

## Abstract

Progressive albuminuria is the primary clinical symptom of diabetic nephropathy (DN), leading to a gradual decline in kidney function. DLX6-AS1 was the first reported long non-coding RNA (lncRNA) to participate in organogenesis and play crucial roles in the brain or neural cell development. Herein, we investigated the DLX6-AS1 (Dlx6-os1 in mice) role in DN pathogenesis. We found that DLX6-AS1 expression in DN patients correlated with the extent of albuminuria. Dlx6-os1 overexpression induced cellular damage and inflammatory responses in cultured podocytes through miR-346-mediated regulation of the GSK-3β pathway. In various established diabetic and newly developed knockout mouse models, Dlx6-os1 knockdown/knockout significantly reduced podocyte injury and albuminuria. The Dlx6-os1 effects were remarkably modulated by miR-346 mimics or mutants and significantly diminished in podocyte-specific GSK-3β-knockout mice. Thus, DLX6-AS1 (Dlx6-os1) promotes DN development by accelerating podocyte injury and inflammation through the upregulation of the GSK-3β pathway, providing a novel molecular target for DN therapy.

## Introduction

Diabetes poses a significant health problem worldwide. It is a systemic, progressive disease-causing damage to multiple organs, diabetic nephropathy (DN) being one of the most common and severe complications [1–4]. The primary clinical symptom of DN is progressive albuminuria [5, 6], but the underlying mechanism still needs to be elucidated. Despite recent advances in DN research, there has been no breakthrough in developing reliable early diagnostic markers or effective treatments for this devastating disease [7–10]. Therefore, there is an unmet medical need to understand the pathogenesis of DN better for facilitating the discovery and development of new targeted therapies. Recent studies have shown that among the various types of kidney tissue damage caused by the diabetic condition, podocyte damage is critical for the development and progression of DN [5, 6, 11, 12]. Inflammatory responses also play essential roles in the development of DN, including damage to podocytes [7, 13–17]. Our laboratory has long been interested in DN pathophysiology and has reported that high glucose (HG)-induced inflammation causes podocyte injury and impairs kidney functions [18–21].

Recently, there has been increasing interest in exploring the role of long non-coding RNA (lncRNA) in the pathogenesis of various chronic diseases, such as cancers, cardiovascular diseases, and immune disorders [2, 22, 23]. LncRNAs are involved in the development and progression of diabetic tissue injury, providing a basis for the identification and evaluation of specific lncRNAs as potential biomarkers for DN [24–28]. Additionally, few reports suggest that lncRNAs have active roles in DN development. For instance, lncRNA SNHG17 regulates Mst1 [26] and the lncRNA PAX8-AS1-N/miR-17-5p/STAT3 axis, affecting podocyte apoptosis [29] and the progression of DN. Yang et al. carried out a high-throughput sequencing study and identified several lncRNA-circRNA-miRNA-mRNA networks associated with type 2 diabetes mellitus (DM) [30]. However, evidence supporting the linkage between lncRNA expression and DN in diabetic patients is lacking. Mechanistic studies on the roles of lncRNAs in diabetic tissue injury might facilitate the development of novel lncRNA-based targeted therapy.

The present study was designed to systematically investigate the role of lncRNAs in the pathogenesis of DN. We first carried out a study to identify specific lncRNAs differentially expressed in DN patients compared with their expression in normal and diabetic patients without kidney injury. The clinical results led to the identification of lncRNA DLX6-AS1 (distal-less homeobox 6 (DLX6) antisense RNA 1, gene ID: 285987) as a major factor associated with the levels of inflammatory factors and renal function in DN patients. DLX6-AS1 (Dlx6-os1 in mice) is found in chromosome 7 in humans (11 splice variants) and chromosome 6 in mice (three splice variants). In both species, DLX6-AS1/Dlx6-os1 contains three exons, exon 3 being the longest. To our knowledge, this is the first report on the role of lncRNA DLX6-AS1 in the pathogenesis and progression of DN. In the present study, we systematically investigated the role of lncRNA DLX6-AS1 using various in vitro and in vivo models with overexpressed or knocked down (KD) lncRNA Dlx6-os1. We also set up a podocyte-specific Dlx6-os1 KO (knockout) mouse model to explore the function of Dlx6-os1 in the podocyte. Furthermore, we carried out mechanistic studies to explore how lncRNA Dlx6-os1 induces podocyte injury, resulting in the discovery of its miR-346-mediated regulation of the GSK-3β pathway, which was confirmed using GSK-3β-KO mice and miR-346 mimics and mutants. GSK-3β plays a critical role in fibroblast differentiation and renal fibrosis [12]. Proximal tubule-specific GSK-3β gene deletion can significantly reduce tubular injury, accelerate regeneration, and suppress renal fibrosis following acute kidney injury in mouse models [31]. Yoshino et al. found that increased levels of GSK-3β can result in severe renal fibrosis in a rat model of unilateral ureteral obstruction [32]. The excessive accumulation of extracellular matrix and overproduction of profibrotic cytokines induced by TGF-β1 treated renal tubular epithelial cells are largely abrogated by GSK-3-β inhibitors [33]. The present results provide a better understanding of the role of lncRNAs in DN pathogenesis, providing a basis for the development of novel approaches to better diagnosis and treatment of albuminuria in DN in the clinic.

## Materials and methods

### Patient specimens

The clinical data, renal tissue sections, and blood and urine specimens were obtained from the Tissue Bank at the First Affiliated Hospital of Zhengzhou University and the National Human Genetic Resources Sharing Service Platform (2005DKA21300). A total of 32 samples (12 DN, 10 DM, and 10 CON) of renal tissues were selected randomly from archived identified biopsy tissue specimens. Normal control kidney tissues were obtained from preimplant biopsy samples or kidneys that were refused to be used in transplantation due to vascular anomalies. The clinical data of all the patients used in the study are shown in Supplementary Table 1. Three random samples from each group were used for lncRNA microarrays (Arraystar 3.0, Aksomics Biotech.Co., Ltd, Shanghai, China), and their corresponding plasma and urine samples were also screened. The selection of target lncRNAs was based on four criteria: (1) the lncRNAs were differentially expressed in the DN patients and CON; (2) the lncRNAs did not show similar differences between DM and CON individuals; (3) the lncRNAs did not show the same expression trends in either the plasma or kidneys in the DN patients; and (4) the lncRNAs showed the same expression trends in both urine samples and kidney tissues of the DN patients. The Institutional Ethics Committee of Zhengzhou University approved this study with an exempt status for human subjects, and the need for informed consent from all participants was waived.

### Animals

The protocols for the use and care of experimental animals were reviewed and approved by the Review Board of Animal Experiments at the University. The National Institutes of Health (NIH) guidelines for animal experiments were followed in this study [34]. The mice were fed at Henan Key Laboratory for Pharmacology of Liver Diseases.

### Mouse type 2 diabetic model

Male 6-week-old type 2 diabetic model db/db and db/m mice (C57BLKS/JGpt, #T002407, GemPharmatech Co., Ltd, China) were allowed to adapt to the animal facility feeding and environmental conditions for 4 weeks and were then injected with lentivirus or saline. db/db mice or db/m mice were randomly allocated into four groups: untreated, saline-treated control (+ saline), shRNA control virus or overexpressed control virus (+ con-shRNA or + con-LV), and lentivirus group with podocyte-specific knockdown or overexpression of lncRNA Dlx6-os1 (+ Dlx6-os1 shRNA or + Dlx6-os1). The animals were sacrificed at 12 weeks after treatment (at the age of 22 weeks). The methods for collecting and analyzing blood, urine, and renal tissue samples were reported previously [11, 19].

### A mouse model with a podocyte-specific knockout of Dlx6-os1

A podocyte-specific lncRNA Dlx6-os1 knockout (Dlx6-os1^flox/flox, nphs2-cre^, Dlx6-os1 KO) mouse model was established (Supplementary Methods and Materials). At 6 weeks of age, the mice were treated with a 45% high-fat diet (MD12032, Medicience Co., Ltd. China) and categorized into groups for subsequent experiments. After 3 weeks of a high-fat diet, the mice received STZ injections. Briefly, male C57BL/6 wild-type mice received intraperitoneal injections of low-dose STZ (55 mg/kg; STZ; Sigma-Aldrich) once a day for 5 consecutive days, during which the mice were still provided with a high-fat diet. The animals were kept fasting for 12 h with access to water before STZ injection. The high-fat diet was replaced with a regular diet following the STZ treatment.

### A mouse model with a podocyte-specific knockout of GSK-3β

Mice were purchased from the Jackson Laboratory (GSK-3βFL, Stock No 029592; NPHS-Cre, Stock No 008205), and the podocyte-specific GSK-3β-KO mice were generated as described previously [35]. The grouping and treatment protocols used in the aforementioned lncRNA Dlx6-os1 overexpression study with the db/m mice were adapted for this experiment.

### Isolation of glomeruli and primary culture of podocytes

The methods used to isolate glomeruli and culture primary podocytes were described in our earlier study [18].

### Renal histology, tissue immunofluorescence, and electron microscopy

As described previously [19], formalin-fixed and paraffin-embedded kidney tissues were processed for histopathological analysis, kidney cortex samples fixed with 2.5% glutaraldehyde were used for EM, and tissue immunofluorescence analyses were carried out with OCT-embedded kidney tissue specimens. In the present study, five random EM fields of glomeruli per mice were examined to count foot processes or measure GBM thickness. These analyses were performed by a single investigator who was blinded to the group assignments.

### Human and mouse kidney RNA fluorescence in situ hybridization (FISH) with immunofluorescence

The Custom LNA™ LncRNA ISH Detection Probes & Kits (QIAGEN, German) was used with a double digoxin-labeled nucleic acid strand probe (human lncRNA *DLX6*-*AS1*: 5DigN/TTGCCTGTTCCATATCAATT/3DigN; mouse lncRNA *Dlx6-os1*: 5DigN/TGTTAAGTGAGACAGGCATTCA/3Dig_N), according to the manufacturer’s instructions.

### Cell culture

MPC5 cells were acquired from ATCC /Shanghai Cell Bank (BLUEFBIO, number BFN60808809). Conditionally immortalized MPC5 cells (passages 5–10) were cultured as reported previously [36, 37]. To determine the extent of podocyte injury and the expression of lncRNA Dlx6-os1 under HG stimulation, differentiated mature mouse podocytes were randomly divided into three groups subjected to different culture conditions: an NG concentration (NG, 5.6 mmol/L D-glucose), hyperosmolar control (HM, 5.6 mmol/L D-glucose + 24.4 mmol/L mannitol), and HG (HG, 30 mmol/L D-glucose). To determine the effects of overexpressing lncRNA Dlx6-os1 on podocytes cultured under NG concentration, cultured normal mouse podocytes were randomly divided into three groups: vehicle control (NG + vehicle), virus control (NG + con-AD), and lncRNA Dlx6-os1- overexpressing adenovirus (NG + Dlx6-os1). To determine the effects of lncRNA Dlx6-os1- knockdown on podocytes cultured with a high concentration of glucose, cultured mouse podocytes were randomly allocated into three groups: vehicle control (HG + vehicle), virus control (HG + con-shRNA), and lncRNA Dlx6-os1-knockdown adenovirus (HG + Dlx6-os1 shRNA).

### RNA-FISH in cultured cells

RNA fluorescence in situ hybridization (FISH) detection of lncRNA Dlx6-os1 in podocytes cultured with different glucose concentrations (NG and HG) was accomplished using a FISH detection kit and probe for lncRNA Dlx6-os1 (C10190 FISH Kit and lnc1100312, Bo Rui Biotech. Co., Ltd, Guangzhou, China) according to the manufacturer’s instructions. The miR-346-5p was detected using Custom miRCURY™ LNA™ microRNA ISH Detection Probes & Kits (339115, QIAGEN).

### Immunofluorescence staining

The cultured podocytes or renal tissue sections were fixed using cold methanol for 40 min and incubated overnight at 4 °C with various primary antibodies (Supplementary Methods and Materials). The secondary antibodies conjugated with Alexa Fluor® 488 or 594 (Invitrogen, Carlsbad, CA, USA) were incubated at 25 °C for 2 h. Then, the sections were counterstained with 4′,6-diamidino-2-phenylindole (DAPI, Vector Laboratories, Burlingame, CA, USA) and visualized under a Zeiss LSM 880 confocal microscope (Carl Zeiss, Oberkochen, Germany). The intensity of the immunofluorescence staining was analyzed in ImageJ software.

### Lentivirus vectors, adenovirus vectors, and RNA interference

The lncRNA Dlx6-os1 (full length)-overexpressing, specific knockdown shRNA, and control adenovirus (Supplementary Methods and Materials) used in this study were designed and synthesized by Hanbio Co., Ltd. (Shanghai, China). Podocytes were seeded in six-well plates at 60–70% confluence and infected with adenovirus following the manufacturer’s instructions (4 × 10^6^ podocytes; MOI = 100). The media was replaced with fresh ones after 6–8 h of viral infection. Mouse miR-346 mimics and mutants were provided by Bo Rui Biotech. Co., Ltd. (Guangzhou, China), and transfected into cells in the presence of Lipofectamine 3000 (Thermo Fisher Scientific, CA, USA). The lentivirus carrying a mouse *Dlx6-os 1* fragment (exon 3 of lncRNA dlx6-os1 containing binding sites for miR-346) and the lentivirus with specific shRNA to induce knockdown were also constructed by Hanbio Co., Ltd. (Shanghai, China). The information on the vectors is listed in the Supplementary Methods and Materials section. The lentivirus was used to treat db/m and db/db mice via tail vein injections (one single injection: virus titer 10^8^ TU/ml; 80–100 µl per mouse). The sequences of the adenovirus and lentivirus packaging fragments are listed in the Supplementary Methods and Materials. Both the overexpression and shRNA lentiviruses carried the promoter for podocin (nphs-2), a podocyte marker protein. The control lentivirus was HA-tagged and also carried the promoter for podocin (nphs-2). The transfection efficiency of lentivirus with a podocyte-specific promoter in the podocytes of glomeruli was analyzed by immunofluorescence staining showing the expression of HA-tag and podocyte marker protein podocin in the OCT-embedded frozen kidney tissues.

### Immunoblotting

The isolated renal glomeruli, primary podocytes, and cultured cells were lysed and homogenized in radio-immunoprecipitation (RIPA) buffer (Millipore) containing protease inhibitors (Millipore). The protein content was quantified, and immunoblotting was carried out as reported previously [20, 38]. The antibodies used are listed in the Supplementary Methods and Materials. The intensity of the band was analyzed in ImageJ software.

### Quantitative real-time polymerase chain reaction (qRT-PCR)

Total RNA was extracted from renal tissue specimens and cultured cells using previously described methods [39, 40]. The Power SYBR Green kit (Invitrogen) and Bio-Rad CFX96 Real-Time system (Bio-Rad, Pleasanton, CA, USA) were used for qRT-PCR. The sequences of PCR primers are shown in the Supplementary Methods and Materials.

### RNA-sequencing

RNA-seq detection was carried out with podocytes after treatment with the lncRNA Dlx6-os1-overexpressing adenovirus under normal culture conditions, and the results were compared with those obtained for the normal controls. The methods used for RNA-seq experiments and data analysis were as described previously [39, 40], and the differentially expressed genes were identified based on statistical analyses (fold of change >1.5, and *P* < 0.05), followed by various data-mining analyses, including correlation analysis, clustering, GO, and KEGG pathway analyses.

### Luciferase reporter assay

LncRNA Dlx6-os1 fragments containing the putative miR-346-binding site were chemically synthesized. The corresponding mutants were created by mutating the lncRNA Dlx6-os1 fragment seed region binding site. A lncRNA Dlx6-os1 wild-type or mutant plasmid and mmu-miR-346 (3p and 5p) or negative control (NC) were transfected into HEK293T cells for 48 h in the presence of Lipofectamine 3000 (Thermo Fisher Scientific). The cells were collected to detect Renilla luciferase activity using a dual-luciferase reporter assay system (Promega, WI, USA) in TD-20/20.

### Statistical analyses

Data and statistical analyses were performed using SPSS V22.0 (IBM Corporation, Armonk, New York, USA). Measurements were taken from distinct samples (different animals or independent experiments). The data are expressed as the mean ± SD. Statistical analysis methods are detailed in the figure legends. The unpaired *t*-test was used to compare two groups, and ordinary one-way or two-way ANOVA was used to compare multiple groups, followed by Dunnett’s multiple comparisons test, Tukey’s multiple comparisons test, or Sidak’s multiple comparisons test, using GraphPad Prism 8.0 software (GraphPad Software, San Diego, CA, USA). Linear regression analysis was used to examine possible relationships between the two parameters. A value of *P* < 0.05 was considered statistically significant.

## Results

### Highly expressed lncRNA DLX6-AS1 in podocytes is positively correlated with albuminuria in DN patients

**Fig. 1:**
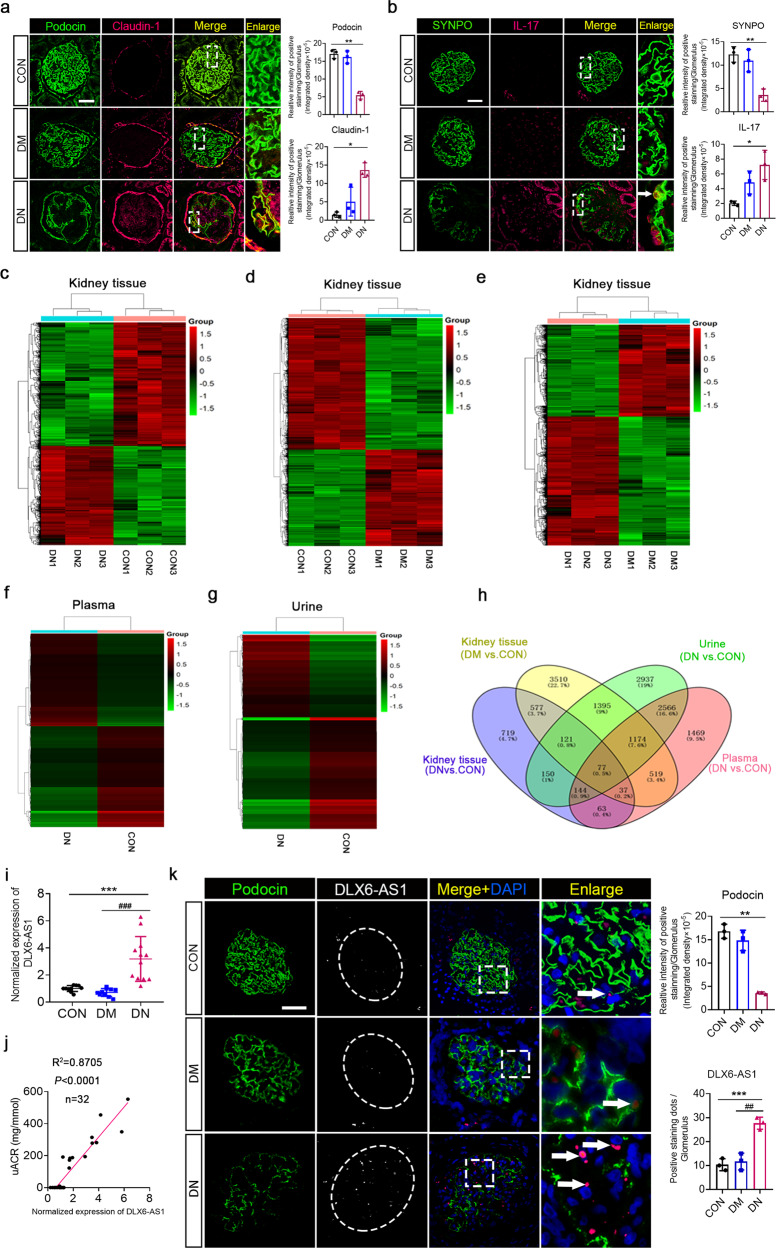
The expression profiles of lncRNAs in the kidney tissue, plasma, and urine samples of diabetic nephropathy (DN) patients, diabetes mellitus (DM) patients, and normal healthy controls (CON). **a** Paraffin-embedded sections from renal biopsies were stained and analysed. Immunofluorescence assays showed a decrease in podocin (podocyte marker protein, green) and an increase in claudin-1 (podocyte injury-associated factor, magenta) DN kidney tissue specimens. The two markers are colocalized (yellow). An enlarged section (dashed rectangle) is shown. The intensity of the staining is quantified and compared in the different samples (*n* = 3). *P < 0.05, **P < 0.01 (unpaired *t*-test). **b** Paraffin-embedded sections from renal biopsies were stained and analysed. Immunofluorescence assays showed a decrease in synaptopodin (SYNPO, podocyte marker protein, green) and an increase in IL-17 (inflammatory factor, magenta) in the DN specimens. The two markers are colocalized (yellow). An enlarged section (dashed rectangle) is shown. The intensity of the staining is quantified and compared in the different samples (*n* = 3). *P < 0.05, **P < 0.01 (unpaired *t*-test). **c**–**g** The lncRNA profiles of the kidney tissue, plasma, and urine samples of the DN patients, diabetic patients, and the CON group participants. **h** A Venn diagram is used to select target lncRNAs. The targets are chosen from among the lncRNAs found to be differentially expressed between the DN patients and CON, excluding those that are also differentially expressed between DM patients and CON. Among these lncRNAs, the same trends of expression in both plasma and kidneys in DN patients are excluded, while those with the same changing trends in urine samples and kidney tissues are preferentially included. Finally, the targets are selected among 150 lncRNAs. **i** qRT-PCR analysis of lncRNA DLX6-AS1 expression in renal tissues showing significant increases in DN patients. ***P < 0.001; ###*P* < 0.001 (unpaired *t-*test). **j** A positive correlation between the uACR and lncRNA DLX6-AS1 expression levels in renal tissues. **k** Paraffin-embedded sections from renal biopsies were stained and analysed. FISH assays show a significant increase in lncRNA DLX6-ASl expression levels in glomeruli of DN patients. DLX6-AS1 staining is shown in grayscale (single channel) or magenta (merge image). White arrows indicate the positions of podocytes with lncRNA DLX6-AS1 expression. Scale bar = 50 µm. **P < 0.01; ***P < 0.001; ##*P* < 0.01 (one-way ANOVA, plus Tukey’s multiple comparisons test).

To investigate the role of lncRNAs in the development and progression of DN, we first determined the pathological changes in renal tissues and lncRNA expression profiles in kidney tissue, plasma, and urine samples of DN and DM patients, and normal healthy controls (CON) (Fig. 1). The pathological changes were analyzed using Periodic acid-Schaff staining (PAS) and electron microscopy (EM) (Supplementary Fig. 1). Increased matrix components, basement membrane thickening, and partial hardening were observed in the kidneys of patients with DN. Similarly, DN kidneys exhibited significant basement membrane thickening and foot process effacement, as observed using EM. Moreover, immunofluorescence assays showed that compared with DM and CON subjects, DN patients experienced a decrease in the expression of podocyte marker proteins, such as podocin and synaptopodin (SYNPO), and an increase in podocyte injury factor claudin-1 and the inflammatory cytokine IL-17 (Fig. 1a, b). These findings indicate that podocyte injury and inflammation might play a key role in the development and progression of DN.

We next performed a high-throughput chip assay to analyze the expression profiles of lncRNAs in the blood, urine, and kidney tissue samples of the participants in the DN, DM, and CON groups. As summarized in Fig. 1c–g, the specific lncRNAs were identified based on comparisons of the expression profiles in DN patients with those in the DM and CON groups. Among 150 differentially expressed lncRNAs (Fig. 1h), five upregulated lncRNAs and four downregulated lncRNAs in DN patients were finally selected for further study based on sequence verification, the availability of specific primers, the fold-change expression (>2.4-fold), and the statistical relevance of the change in expression (*P* < 0.05; compared with that of the CON group). After further confirmation by qRT-PCR, lncRNA DLX6-AS1 was found to be one of the most significantly upregulated lncRNAs in the renal tissue of DN patients (Fig. 1i). Notably, there was a positive correlation between the lncRNA DLX6-AS1 expression level in renal tissues and the urinary albumin/creatinine ratio (uACR) in DN patients (Fig. 1j). Moreover, significantly upregulated lncRNA DLX6-AS1 expression in podocytes accompanied by the decreased expression of podocyte marker protein podocin was found through fluorescence in situ hybridization (FISH) with renal tissues from DN patients (Fig. 1k). These findings suggest that increased lncRNA DLX6-AS1 in podocytes might positively correlate with albuminuria and podocyte injury in DN patients.

### LncRNA Dlx6-os1 is upregulated in the glomeruli of diabetic db/db mice

**Fig. 2:**
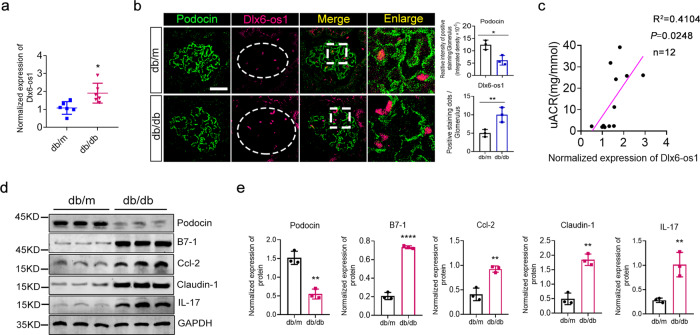
A correlation between increased lncRNA Dlx6-os1 expression and increased podocyte injury in the db/db mice model with diabetic nephropathy. **a** qRT-PCR assays showing a significant increase in lncRNA Dlx6-os1 expression in isolated podocytes from mouse kidney. *P < 0.05 vs. db/m (unpaired *t*-test). *n* = 6 mice/group. **b** FISH-based detection of the expression level and location of lncRNA Dlx6-os1 in paraffin-embedded db/db mouse kidney. The expression of lncRNA Dlx6-os1 in podocyte of db/db mice is upregulated (magenta dots increased), and the expression of podocyte marker protein podocin (green) was reduced. The intensity of the staining is quantified and compared in the different samples (*n* = 3). *P < 0.05, **P < 0.01, (unpaired *t*-test). Scale bar = 20 µm. Three visual fields were analyzed per sample. *n* = 3 mice/group. **c** Correlation between uACR and lncRNA Dlx6-os1 expression levels in isolated podocyte from db/db and db/m mouse kidney. **d** Western blots of podocin, B7-1, ccl-2, IL-17, and claudin-1 in isolated glomeruli from mouse kidney. GAPDH was used as the loading control. **e** Quantification from the western blot data shown in **d**. The intensity of the bands was analyzed in ImageJ and normalized to the intensity of GAPDH (*n* = 3). **P < 0.01, ****P < 0.0001 vs. db/m (unpaired *t*-test).

To demonstrate the role of lncRNA DLX6-AS1 in DN development, we employed diabetic mice models. We used db/db mice as a type 2 diabetic mice model and db/m mice as a non-diabetic control to observe the correlations between the lncRNA Dlx6-os1 expression level, urinary albumin protein level, and the degree of podocyte injury. Groups of diabetic db/db mice were sacrificed at 14 and 22 weeks of age, and tissues were collected for analysis (Supplementary Fig. 2a). As expected, for all age groups, the db/db mice displayed a significantly higher uACR and higher random blood glucose (RBG) levels (*P* < 0.001, Supplementary Fig. 2b, c) and a lower kidney weight/body weight ratio (KW/BW, *P* < 0.01, Supplementary Fig. 2d). PAS staining of kidney tissues showed significantly increased thickening of the renal basement membrane in 14- and 22-week-old db/db mice compared with db/m mice. Similarly, electron microscopy showed basement membrane thickening and podocyte foot process effacement (Supplementary Fig. 2e). Interestingly, Dlx6-os1 expression in the glomeruli of the db/db mice increased significantly (Fig. 2a, b), while podocin expression decreased (Fig. 2b). Moreover, similar to the above findings in human DN patients, the expression of Dlx6-os1 in the glomeruli positively correlated with uACR in the db/db mice (Fig. 2c). In addition, the levels of inflammatory and injury-associated factors significantly increased in the podocytes of db/db mice compared with those in the podocytes of db/m mice (Fig. 2d, e). The decrease of podocin expression detected in the microscopy samples (Fig. 2b) was further verified by Western blot (WB) (Fig. 2d, e), indicating that Dlx6-os1 expression was closely associated with increased podocyte injury.

### LncRNA Dlx6-os1 overexpression promotes podocyte injury in db/m mice

**Fig. 3:**
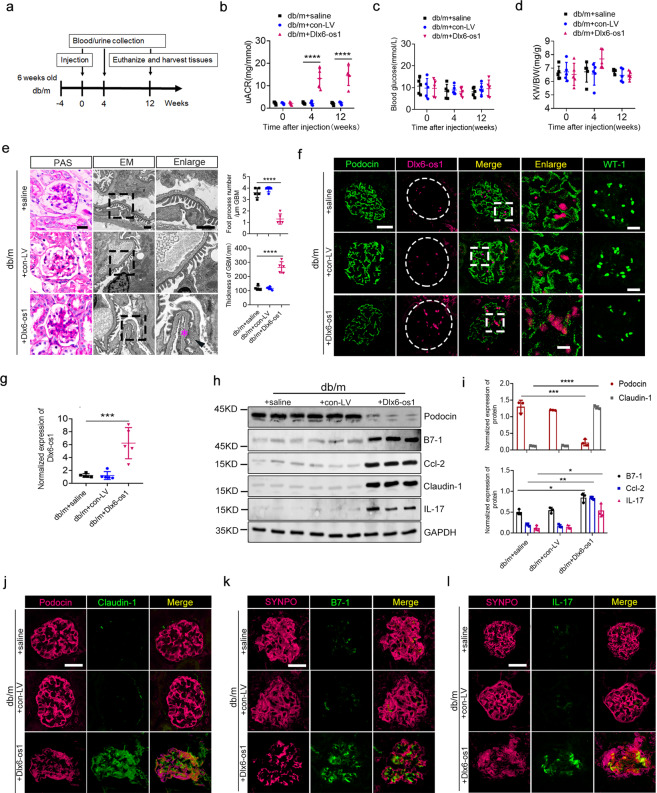
Glomerular podocyte injury induced by lncRNA Dlx6-os1 overexpression in phenotypically normal db/m mice. **a** Treatment flow chart. Mice were on an adaptive feeding for 4 weeks before the experiment (−4 to 0 weeks). The db/m mice were randomly allocated to three groups: saline control (db/m + saline), blank virus control (db/m + con-LV), and lncRNA Dlx6-os1-overexpressing lentivirus (db/m + Dlx6-os1) groups. **b**–**d** The uACR of the (db/m + Dlx6-os1) mice is significantly higher than that of the (db/m + saline) mice (**b**), but there are no significant differences in the random blood glucose level (**c**) or kidney weight/ body weight (KW/BW) ratio (**d**). ****P < 0.0001. *n* = 5 mice/group (one-way ANOVA plus Dunnett’s multiple comparisons test). **e** Periodic acid-Schiff (PAS) staining and electron microscopy (EM) analysis of kidney tissues and podocytes from db/m mice 12 weeks after lentivirus injection. The statistical charts show the number of foot processes per micrometer and the thickness of the basement membrane. Five visual fields were analyzed per sample, PAS bar = 20 µm; EM bar = 1 µm). ****P < 0.0001 *(*unpaired *t*-test). *n* = 5 mice/group. **f** FISH-based detection of the location and level of the lncRNA Dlx6-os1 expression in paraffin-embedded mouse kidney tissues. LncRNA Dlx6-os1 is upregulated (magenta dots). Podocin (green) and WT-1 expressions are reduced. Images are representatives of three separate experiments. Scale bar = 20 µm. **g** The expression of lncRNA Dlx6-os1 in the primarily isolated podocyte as determined by qRT-PCR. ***P < 0.001 (unpaired *t*-test). *n* = 5 mice/group. **h** Western blots of isolated glomeruli from mouse kidneys to determine the levels of podocin, B7-1, ccl-2, IL-17, and claudin-1 protein. GAPDH was used as the loading control. *n* = 3 mice/group. **i** Quantification from the western blot data shown in **h**. The intensity of the bands was analyzed in ImageJ and normalized to that of GAPDH (*n* = 3). *P < 0.05, **P < 0.01, ***P < 0.001, ****P < 0.0001 (one-way ANOVA, plus Tukey’s multiple comparisons test). **j**–**l** Comparison of podocin (**j**, magenta), SYNPO (synaptopodin, **k** and **l**, magenta), claudin-1 (**j**, green), B7-1 (**k**, green), and IL-17 (**l**, green) expression levels and localization sites in OCT-embedded frozen kidney tissues. Images are representative of three separate experiments. Scale bar = 20 µm.

To investigate whether lncRNA Dlx6-os1 overexpression could induce pathogenic effects, 10-week-old db/m mice were injected with the lentivirus overexpressed lncRNA Dlx6-os1 (the exon 3 of Dlx6-os1) for 12 weeks (Fig. 3a). The transduction efficiency was shown in Supplementary Fig. 3. Dlx6-os1 overexpression led to significantly increased uACR at 12 weeks post-injection (*P* < 0.0001, Fig. 3b) but no remarkable changes in the random blood glucose levels or the KW/BW (Fig. 3c, d). In addition, PAS staining showed increased renal tissue matrix and thickened basement membrane, and EM revealed evident thickening of the basement membrane and podocyte foot process effacement (*P* < 0.0001, Fig. 3e). The overexpression of Dlx6-os1 in podocytes was confirmed by FISH (Fig. 3f) and qRT-PCR (Fig. 3g). As the expression of lncRNA Dlx6-os1 gradually increased in the mouse glomeruli, the expression of podocyte marker proteins gradually decreased (Fig. 3h–l). WT-1, expressed in the podocyte nucleus, was also found to decrease after lncRNA Dlx6-os1 overexpression (Fig. 3f), which indicated the loss number of podocytes. Notably, the expression of inflammatory and injury-associated factors also increased in these podocytes (Fig. 3h–l). The intensity of the immunofluorescence staining were analyzed and shown in Supplementary Fig. 4. These results demonstrated that increased lncRNA Dlx6-os1 expression promoted podocyte injury, inflammation, and albuminuria in db/m mice.

### Knockdown of lncRNA Dlx6-os1 alleviates podocyte injury in diabetic mice

**Fig. 4:**
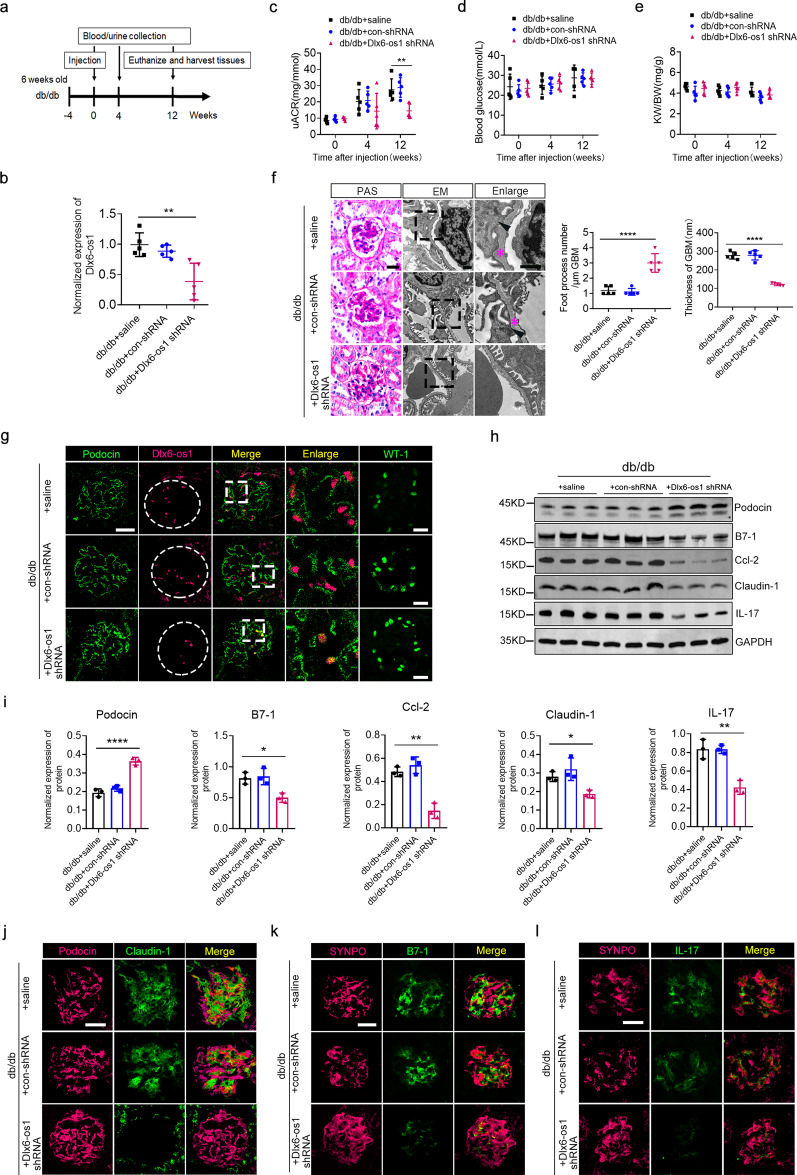
Effects of lncRNA Dlx6-os1 knockdown on podocyte injury, inflammation, and proteinuria in db/db mice. **a** Treatment flow chart. The 10-week-old db/db mice were treated with lncRNA Dlx6-os1 shRNA; then, blood, urine, and kidney tissue samples were analyzed at 4- and 12- weeks post-injection. **b** qRT-PCR assays showing that lncRNA Dlx6-os1 expression is significantly reduced in the shRNA-treated db/db mice compared with that of the controls in isolated podocytes from mouse kidney. **P < 0.01 (unpaired *t*-test). *n* = 5 mice/group. **c**–**e** The mice were injected with saline (db/db + saline), blank lentivirus (db/db + con-shRNA), or lncRNA Dlx6-os1 shRNA lentivirus (db/db + Dlx6-os-shRNA). The urinary albumin/creatinine ratio (uACR), random blood glucose, and kidney weight/body weight (KW/BW) ratio were compared among the three groups at different times after injection. **P < 0.01 (one-way ANOVA plus Dunnett’s multiple comparisons test). *n* = 5 mice/group. **f** Periodic acid-Schiff (PAS) staining and electron microscopy (EM) analysis of podocyte injury in kidney tissues 12 weeks after treatment with lncRNA Dlx6-os1 shRNA lentivirus. The below statistical graphs show the number of foot processes per micrometer and the basement membrane thickness of the db/db mice under different treatments. Five observation fields are collected per sample, PAS bar = 20 µm; EM bar = 1 µm. ****P < 0.0001 (unpaired *t*-test). *n* = 5 mice/group. **g** FISH analysis in paraffin-embedded kidney tissue samples. The lncRNA Dlx6-os1 shRNA-treated mice show decreased lncRNA Dlx6-os1 expression in podocytes (magenta dots) and increased podocin (green) and WT-1 expressions (green) (*n* = 3). Scale bar = 20 µm. **h** Western blot to detect the expression levels of podocin, B7-1, ccl-2, IL-17, and claudin-1 of isolated glomeruli from mouse kidney. GAPDH was used as the loading control. *n* = 3 mice/group. **i** Quantification from the western blot data shown in **h**. The intensity of the bands was analyzed in ImageJ and normalized to that of GAPDH (*n* = 3). *P < 0.05, **P < 0.01, ***P < 0.001, ****P < 0.0001 (one-way ANOVA, plus Dunnett’s multiple comparisons test). **j**–**l** Comparison of podocin (**j**, magenta), SYNPO (synaptopodin, **k** and **l**, magenta), claudin-1 (**j**, green), B7-1 (**k**, green), and IL-17 (**l**, green) expression levels and localization sites in OCT-embedded frozen kidney tissues (*n* = 3). Scale bar = 20 µm.

We next observed the effects of lncRNA Dlx6-os1 knockdown (KD) on podocyte injury, inflammation, and albuminuria in db/db mice. Ten-week-old db/db mice were injected with lentivirus-lncRNA Dlx6-os1 shRNA (Fig. 4a), and Dlx6-os1 reduction was confirmed by qRT-PCR in isolated primary podocyte at 12 weeks after injection (*P* < 0.01, Fig. 4b). Dlx6-os1 KD in podocytes resulted in significantly lower uACR levels after 12 weeks (*P* < 0.01, Fig. 4c), but no changes were detected in the RBG level or KW/BW ratios (Fig. 4d, e). An increase in the foot process number and a decrease in the thickness of GBM, measured using PAS staining and EM, indicated significantly reduced podocyte injury in the lncRNA Dlx6-os1 KD mice (*P* < 0.0001, Fig. 4f). Notably, podocin, WT-1, and SYNPO expression gradually increased, accompanied by the decreased expression of lncRNA Dlx6-os1 (Fig. 4g–l), whereas the inflammatory and injury-associated factors in the podocytes were reduced in the lncRNA Dlx6-os1 KD mice (Fig. 4h–l). The intensity of the immunofluorescence staining were analyzed and shown in Supplementary Fig. 5. Taken together, these results suggested that inhibiting lncRNA Dlx6-os1 expression in kidney podocytes could represent an effective approach to alleviate the podocyte injury, inflammation, and albuminuria associated with DN.

### Podocyte-specific lncRNA Dlx6-os1 knockout remarkably reduces the development of diabetic nephropathy in the streptozotocin (STZ)-induced diabetic mice

**Fig. 5:**
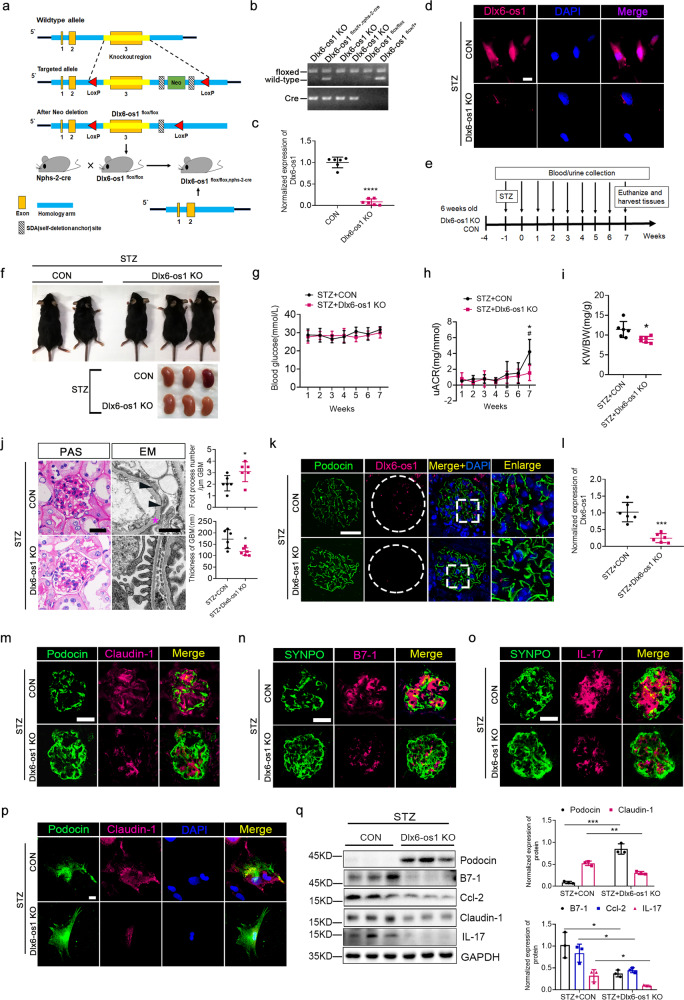
Establishment of a podocyte-specific lncRNA Dlx6-os1-knockout (KO) mouse model and development of streptozotocin (STZ)-induced diabetic kidney injury. **a** A flow chart showing the preparation of lncRNA Dlx6-os1-specific knockout mice. **b** Verification of gene knockout in knockout mice (Dlx6-os1 KO). Both floxed and cre bands are positive, while the negative results for the wild-type gene band confirmed podocyte-specific knockout of lncRNA Dlx6-os1. **c** qRT-PCR analysis of primary cultured podocytes isolated from the glomeruli of the knockout mice, compared to the control flox/flox mice, confirms the lack of lncRNA Dlx6-os1. ****P < 0.0001 vs. CON (unpaired *t*-test). *n* = 6 mice/group. **d** FISH on primary podocytes isolated from STZ-treated Dlx6-os1 KO mice and STZ-treated control mice (*n* = 3). Scale bar = 20 µm. **e** Flow chart showing the STZ-mediated induction of diabetes in Dlx6-os1 KO mice. Six-week-old male Dlx6-os1 KO mice or CON (flox/flox) mice were fed a high-fat diet. After given intraperitoneal injections of STZ (55 mg/kg, once a day) for five consecutive days, blood and urine samples were collected weekly; animals were sacrificed 7 weeks after the STZ injection. *n* = 6 mice/group. **f** No significant differences in the appearance of the control (CON; flox/flox) and KO mice after STZ induction is observed, although the control mice had larger kidneys than the KO mice. **g** No significant difference is observed in the random blood glucose levels between the two groups of mice with diabetes induced by STZ, and both groups have levels significantly higher than the level of the flox/flox control mice (Supplementary Fig. 3a). **h** Increase in the urinary albumin/creatinine ratio (uACR) levels in the STZ-CON mice 5 weeks after injection. *P < 0.05 (7-week STZ + CON) vs. (1-week STZ + CON), #*P*  < 0.05 vs. (STZ + CON*)* at the same age (two-way ANOVA, plus Dunnett’s multiple comparisons test or Sidak’s multiple comparisons test). **i** Kidney weight/body weight (KW/BW) ratio in the wild-type (STZ-CON) and KO mice (STZ-Dlx6-os1 KO) after STZ induction. *P < 0.05 vs. (STZ + CON) (unpaired *t*-test). *n* = 6 mice/group. **j** Periodic acid-Schiff (PAS) staining and electron microscopy (EM) of kidney tissues revealed reduced damage in the kidney structures of the Dlx6-os1-KO mice following STZ treatment. The statistical chart compares the number of podocyte foot processes per micrometer and the thickness of the basement membrane between the two groups. *P < 0.05 vs. (STZ + CON) (unpaired *t*-test). *P*AS bar = 20 µm; EM bar = 1 µm. *n* = 6 mice/group. **k** FISH showing almost no expression of lncRNA Dlx6-os1 in the knockout mouse glomeruli. FISH bar = 20 µm. Images are representatives of three separate experiments. **l** qRT-PCR used to detect lncRNA Dlx6-os1 expression in primary cultured podocytes isolated from the glomeruli of knockout and control (flox/flox) mice after STZ induction. ***P < 0.001 vs (STZ + CON) (unpaired *t*-test). *n* = 6 mice/group. **m**–**o** Immunofluorescence analyses of (**m**) injury-related factors (claudin**-**1, magenta), (**n**, **o**) inflammatory factors (B7-1 and IL-17, in magenta) and podocyte marker proteins (**m**: podocin, green; **n**, **o**: SYNPO, synaptopodin, green) in OCT-embedded frozen kidney tissues. Podocyte-specific lncRNA Dlx6-os1 knockout mice show less extensive injury and inflammation. Scale bar = 20 µm. Images are representatives of three separate experiments. **p** Immunofluorescence analyses of claudin-1 (magenta) and podocin (green) in primary cultured podocytes isolated from the glomeruli of knockout and control (flox/flox) mice after STZ induction. Scale bar = 20 µm. Images are representatives of three separate experiments. **q** Western blots of podocyte marker proteins, injury-associated factors, and inflammatory factors of isolated glomeruli from mouse kidney. STZ-induced renal podocyte damage is significantly alleviated in knockout mice. Quantification of the western blot data is shown in the right panels. The intensity of the bands was analyzed in ImageJ and normalized to that of GAPDH (*n* = 3). *P < 0.05, **P < 0.01, ***P < 0.001 (unpaired *t*-test). *n* = 3 mice/group.

We next established a podocyte-specific lncRNA Dlx6-os1 knockout (Dlx6-os1 KO) mouse model (Dlx6-os1^flox/flox, nphs2-cre^) and confirmed the knockdown of Dlx6-os1 in these mice by qRT-PCR (Fig. 5a–c). In addition, we confirmed the expression and localization of Dlx6-os1 using FISH in primary podocytes isolated from an STZ-induced control mouse and an STZ-induced Dlx6-os1 KO mouse (Fig. 5d). When measuring the RBG, uACR, or KW/BW ratio between the lncRNA Dlx6-os1 KO mice and the control mice (flox/flox), we found no significant differences in non-diabetic condition (Supplementary Fig. 6a–c). Thus, we induced diabetes in both lncRNA Dlx6-os1 KO mice and control mice by the standard STZ induction method (Fig. 5e, f) and high-fat (45% fat content) diet. Compared to the non-diabetic mice, the RBG levels were elevated in both the lncRNA Dlx6-os1 KO mice and flox/flox control mice one week after STZ injection (Fig. 5g compared to Supplementary Fig. 6a). The uACR levels in the STZ-induced diabetic control mice were significantly increased compared with lncRNA Dlx6-os1 KO mice after STZ injection at 7 weeks (Fig. 5h; *P* < 0.05). However, the KW/BW ratio of STZ-induced lncRNA Dlx6-os1 KO mice was significantly lower than that of STZ-induced control mice at 7 weeks after injection (*P* < 0.05, Fig. 5i). Using PAS staining and EM, glomerular matrix deposition and basement membrane thickness were found to reduce, and foot process effacement was also found to decrease in the diabetic lncRNA Dlx6-os1 KO mice (Fig. 5j), suggesting a protective role. Moreover, the expression of lncRNA Dlx6-os1 in glomeruli podocytes of lncRNA Dlx6-os1 KO mice was significantly reduced even under the STZ treatment (*P* < 0.001, Fig. 5k, l), and the STZ-induced lncRNA Dlx6-os1 KO mice showed increased expression of glomerular podocyte marker proteins and reduced levels of inflammatory factors and podocyte injury-associated proteins (Fig. 5m–q). The intensity of the immunofluorescence staining were analyzed and shown in Supplementary Fig. 7. Therefore, the results indicate that Dlx6-os1 knockout could mitigate the development of diabetic nephropathy in mice.

### LncRNA Dlx6-os1 mediates podocyte injury and inflammation in vitro

**Fig. 6:**
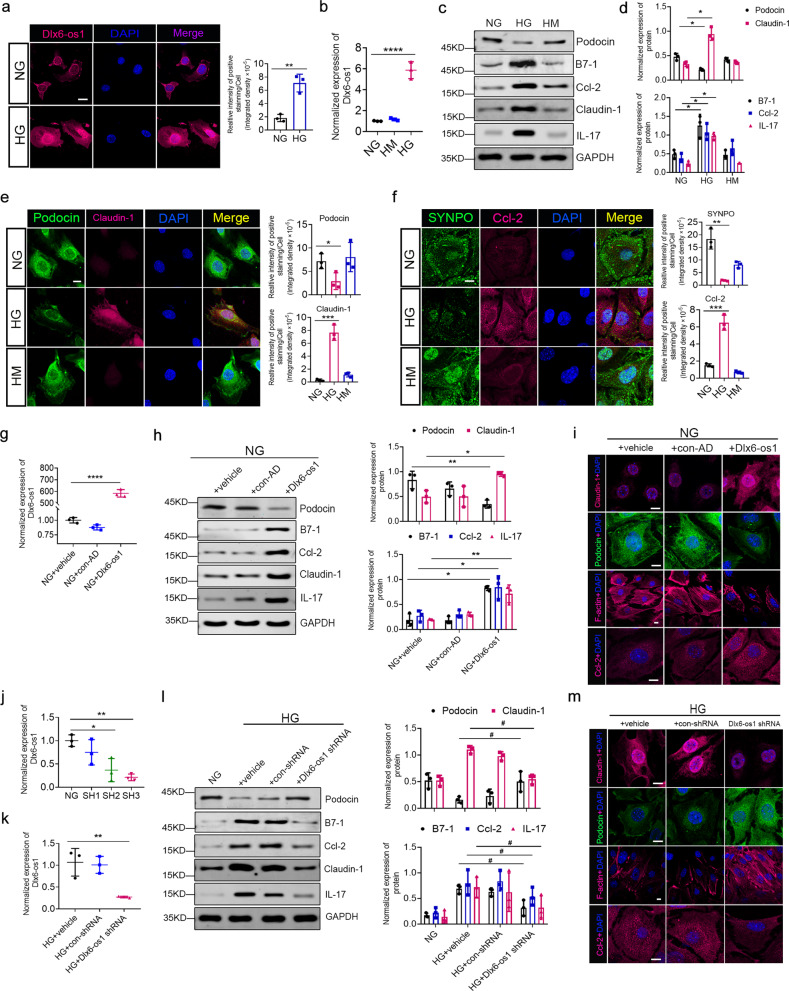
LncRNA Dlx6-os1 expression level, localization, and its relationship with podocyte injury in cultured cells. **a** FISH analysis of lncRNA Dlx6-os1 expression and colocalization with DAPI in the mouse podocyte cell line MPC5 cultured under normal glucose (NG, 5.6 mM) and high glucose (HG, 30 mM) conditions. Images are representatives of three separate experiments. The intensity of the staining is quantified and compared in the different samples (*n* = 3). **P < 0.01, (unpaired *t*-test). **b** The expression of lncRNA Dlx6-os1 in MPC5 cells as determined by qRT-PCR. NG (5.6 mM), HG (30 mM) and HM (5.6 mM glucose and 24.4 mM mannitol) conditions. ****P < 0.0001 (unpaired *t*-test). *n* = 3 independent experiments. **c** Western blot of podo**c**yte marker protein (podocin), injury-associated factors (claudin-1), and inflammatory factors (B7-1, IL-17, and ccl-2) of MPC5 podocytes under different glucose treatments. **d** Quantification of the western blot data shown in **c**. The intensity of the bands was analyzed in ImageJ and normalized to that of GAPDH (*n* = 3 independent experiments). *P < 0.05 (Two-way ANOVA plus Dunnett’s multiple comparisons test). **e**, **f** Immunofluorescence-based detection of the podocyte marker proteins podocin (**e**, green) and SYNPO (synaptopodin, **f**, green) and markers of podocyte injury and inflammation (claudin-1, **e**, magenta and ccl-2, **f**, magenta) in MPC5 cells. Images are representatives of three separate experiments. **g** qRT-PCR analysis of lncRNA Dlx6-os1 expression in the cells treated with vehicle, con-AD (empty adenovirus), or Dlx6-os1 (overexpression lncRNA Dlx6-os1 adenovirus) in MPC5 cells. ****P < 0.0001 (unpaired *t*-test). *n* = 3. **h** Western blots of the podocyte marker protein podocin; cell damage factor claudin-1; inflammatory factors B7-1, ccl-2, and IL-17 of MPC5 cells (vehicle, con-AD or cells overexpressing Dlx6-os1) under NG conditions. Quantification of the western blot data were shown on the right. The intensity of the bands was analyzed in ImageJ and normalized to that of GAPDH (*n* = 3). *P < 0.05; **P < 0.01 (Two-way ANOVA plus Dunnett’s multiple comparisons test). **i** Immunofluorescence-based detection of podocyte morphology evaluated by the expression and localization of F-actin, claudin-1, and podocin in MPC5 cells (vehicle, con-AD or cells overexpressing Dlx6-os1). **j** qRT-PCR analysis to show the efficiency of various shRNAs (SH1-3) on lncRNA Dlx6-os1 targeting. MPC5 cells (NG condition) were used. SH3 showing the best interference and used in subsequent studies. *P < 0.05 (unpaired *t*-test). *n* = 3. **k** qRT-PCR results for lncRNA Dlx6*-*os1 expression levels after SH3 treatment and culturing under HG conditions (HG + Dlx6-os1 shRNA). ***P < 0.001 (unpaired *t*-test). *n* = 3. **l** Western blots of podocyte marker proteins, injury-associated factors, and inflammatory factors of MPC5 cells. Quantification of the western blot data is shown on the right. The intensity of the bands was analyzed in ImageJ and normalized to that of GAPDH (*n* = 3). #*P* < 0.05 (Two-way ANOVA plus Dunnett’s multiple comparisons test). **m** Immunofluorescence-based analysis of the morphological changes of podocytes and a podocyte marker protein (podocin, green), podocyte injury-associated factor (claudin-1, magenta), inflammatory cytokine (ccl-2, magenta), and a cytoskeletal protein (F-actin, magenta) in MPC5 cells cultured under HG conditions following lncRNA Dlx6-os1 shRNA treatment. Scale bar = 10 µm in **a**, **b**, **e**, **f**, **i**, **m**.

Next, a mouse podocyte cell line (MPC5) was used for further assessing the role of the lncRNA Dlx6-os1 in podocyte injury. MPC5 cells were cultured under normal glucose (NG), hyperosmolar (HM), and HG (high glucose) conditions. The HG condition exhibited higher levels of the lncRNA Dlx6-os1 in both the nucleus and cytoplasm of the cells (*P* < 0.0001, Fig. 6a, b). MPC5 cells under HG treatment also showed lower levels of podocyte marker proteins, including podocin (Fig. 6c–e) and SYNPO (Fig. 6f), and higher levels of injury-associated claudin-1 (Fig. 6c–e) and inflammatory factors such as B7-1, IL-17 (Fig. 6c, d), and ccl-2 (Fig. 6c–f). These findings indicated that HG led to the upregulation and re-localization of the lncRNA Dlx6-os1, possibly leading to podocyte injury and inflammation. Moreover, adenovirus-induced overexpression of the full-length of lncRNA Dlx6-os1 in MPC5 cells under NG conditions (*P* < 0.0001, Fig. 6g) resulted in reduced expression of podocyte marker proteins, increased levels of inflammatory factors and injury-associated factors, and damage to the cytoskeleton (Fig. 6h, i). To evaluate the specificity of Dlx6-os1 overexpression in these processes, we next attempted to reverse these phenomena. We tested the effect of three Dlx6-os1 shRNA under NG conditions (Fig. 6j), and SH3 was selected for further experiments based on the silencing efficacy. Our data showed that HG-induced Dlx6-os1 upregulation could be reverted using shRNA (Fig. 6k), while inflammatory and injury-associated factors could decrease in consequence (Fig. 6l, m). Together, these results further support the role of the lncRNA Dlx6-os1 in podocyte injury.

### LncRNA Dlx6-os1 regulates GSK-3β expression and signaling

**Fig. 7:**
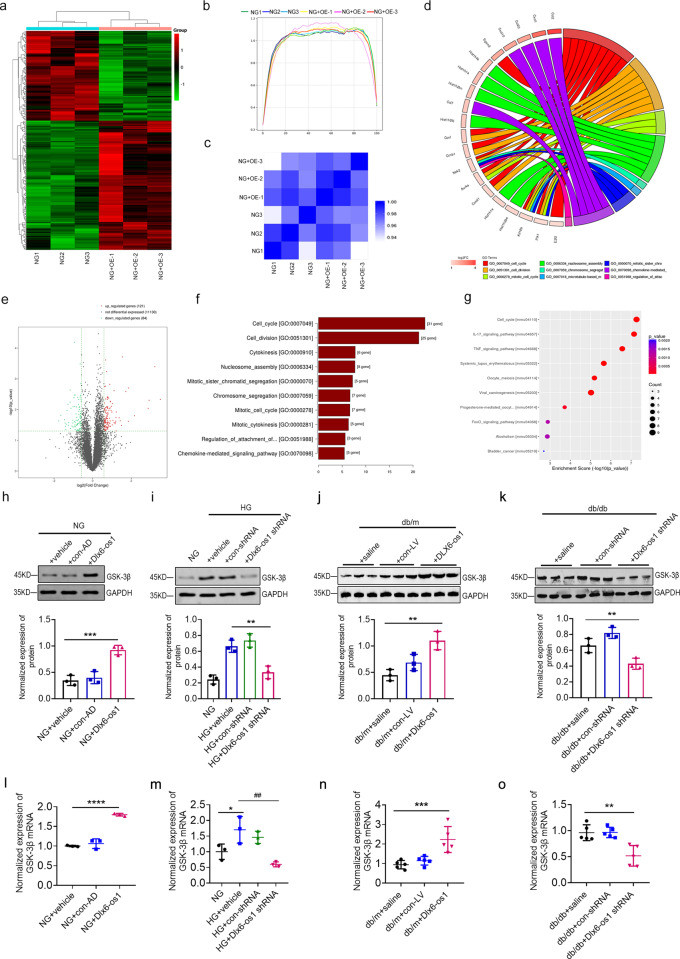
RNA-seq results after lncRNA Dlx6-os1 overexpression in the cultured podocytes. **a** The RNA-seq and cluster analysis. NG, normal cultured podocytes; NG + OE, lncRNA Dlx6-os1-overexpressing podocytes. **b** Transcription coverage map. **c** A correlation coefficient heat map of samples. The shade of blue represents the degree of correlation. **d** The circle map of differential expression mRNA. **e** A differential expression mRNA volcano plot. **f** The BP-enrichment score chart. **g** The pathway analysis obtained for the enrichment score dot plot. The top ten pathways are shown. **h**–**k** Western blots of the GSK-3β protein of both cultured podocytes (MPC5) and isolated glomeruli from db/m and db/db mice under different treatments. Quantification of the western blot data were shown below. The intensity of the bands was analyzed in ImageJ and normalized to that of GAPDH (*n* = 3). **P < 0.01, ***P < 0.001 (one-way ANOVA plus Dunnett’s multiple comparisons test). **l**–**o** qRT-PCR analysis of GSK-3β gene expression in both cultured podocytes (MPC5, *n* = 3) and primary podocytes isolated from db/m and db/db mice under different treatments (*n* = 5). *P < 0.05, **P < 0.01, ***P < 0.001, ****P < 0.0001; ##*P* < 0.01 (one-way ANOVA plus Dunnett’s multiple comparisons test).

To further explore the mechanisms of podocyte injury caused by lncRNA Dlx6-os1, we performed RNA-sequencing with MPC5 cells overexpressing lncRNA Dlx6-os1 and control cells. As shown in Fig. 7a–e, compared with the control group, several differentially expressed mRNAs were identified in the MPC5 cells overexpressing lncRNA Dlx6-os1. Gene Ontology (GO) and Kyoto Encyclopedia of Genes and Genomes (KEGG) pathway analyses showed that the most significantly changed pathways were found in the cell cycle-regulating (Fig. 7f) and IL-17 signaling pathways (Fig. 7g). Interestingly, GSK-3β was involved in both pathways (Supplementary Fig. 8a, b), and it has been described to play a nonignorable role in several diseases [35], including diabetic nephropathy and glomerular podocyte injury [18, 20]. Hence, we evaluated GSK-3β expression by WB and qRT-PCR in MPC5 podocytes cultured under NG (Fig. 7h, l) and HG (Fig. 7i, m) conditions and isolated glomeruli from db/m (Fig. 7j, n) and db/db (Fig. 7k, o) mice receiving different treatments. These results indicated a correlation between Dlx6-os1 and GSK-3β expression, suggesting that the lncRNA Dlx6-os1 could exert its effects at least partly by regulating the GSK-3β signaling pathway.

### LncRNA Dlx6-os1 triggers podocyte injury by regulating the miR-346-mediated GSK-3β pathway

**Fig. 8:**
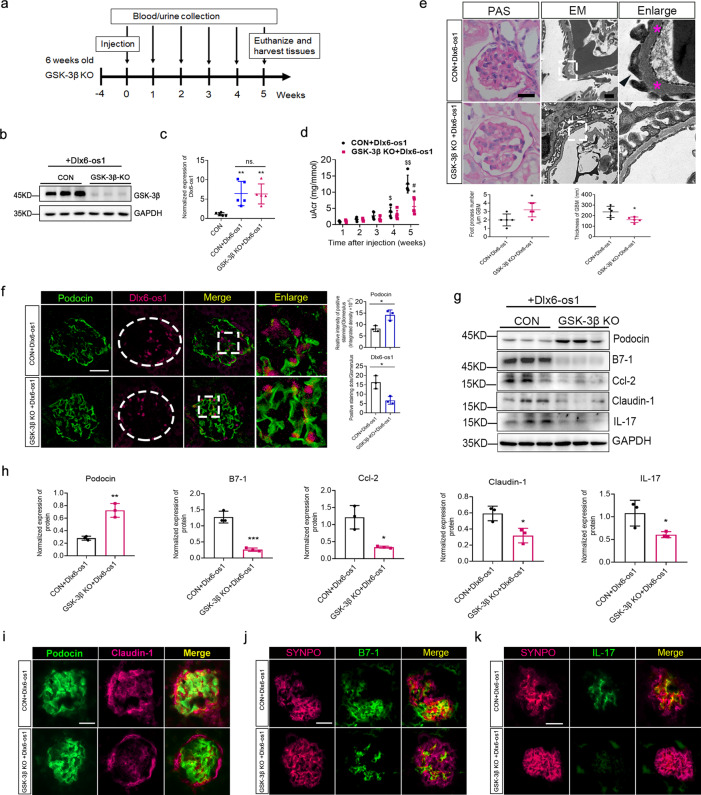
LncRNA Dlx6-os1 inducing glomerular podocyte injury by regulating the GSK-3β pathway, confirmed using podocyte-specific GSK-3β-knockout (cKO) mice. **a** Flow chart of the model establishment, monitoring, and sampling. **b** Comparison of the GSK-3β protein expression levels in glomeruli isolated from control (flox/flox) or GSK-3β-KO mice. **c** Expression of lncRNA Dlx6-os1 in glomeruli of CON or GSK-3β-KO mice injected with lncRNA Dlx6-os1-overexpression-lentivirus 5 weeks after injection assessed using qRT-PCR, compared to non-injected mice. **P < 0.01 vs. CON; ns no significant difference (one-way ANOVA plus Dunnett’s multiple comparisons test). *n* = 5. **d** A comparison of the urinary albumin/creatinine ratio (uACR) values. $*P* < 0.05 vs. (1-week CON + Dlx6-os1); $$*P* < 0.01 vs. (1-week CON + Dlx6-os1); #*P* < 0.05 vs. (1-week GSK-3β-KO + Dlx6-os1); *P < 0.05 vs. (5-week CON + Dlx6-os1). Two-way ANOVA plus Dunnett’s multiple comparisons test or Sidak’s multiple comparisons test (*n* = 5 mice per group). **e** Periodic acid-Schiff (PAS) staining and electron microscopy (EM) showed improved pathology in the kidney tissues of GSK-3β-KO mice, compared with the flox/flox control. PAS bar = 20 µm; EM bar = 1 µm. Quantification is shown below the images (*n* = 5). *P < 0.05 vs. (CON + Dlx6-os1) (unpaired *t*-test). **f** FISH detecting the expression and localization of lncRNA Dlx6-os1 (magenta), and immunofluorescence detecting podocin expression (green) in glomeruli of control and GSK-3β-KO mice overexpressing Dlx6-os1. Images are representatives of three separate experiments. Bar = 20 µm. The intensity of the staining is quantified and compared in the different samples (*n* = 3). *P < 0.05 (unpaired *t*-test). **g** Western blots of isolated glomeruli to detect the expression of podocyte markers (podocin), injury-associated protein claudin-1, and inflammatory factors in the lncRNA Dlx6-os1 overexpressed GSK-3β-KO mice and control (CON) mice. Blots are representatives of three separate experiments and quantification is shown in **h**. **h** Quantification of the western blot data shown in **g**. The intensity of the bands was analyzed in ImageJ and normalized to that of GAPDH (*n* = 3). *P < 0.05 (vs CON + Dlx6-os1), **P < 0.01 (vs CON + Dlx6-os1), ***P < 0.01 (vs CON + Dlx6-os1) (unpaired *t*-test). **i**–**k** Immunofluorescence detecting the expression and localization of podocyte marker proteins (podocin, **i**, green; SYNPO, synaptopodin **j**, and **k**, magenta), injury protein claudin-1 (**i**, magenta,) and inflammatory factors (**j**, B7-1 green and **k**, IL-17, green) in glomeruli of control and GSK-3β-KO mice overexpressing Dlx6-os1. Bar = 20 µm.

Hence, we sought to determine whether lncRNA Dlx6-os1 could induce podocyte injury and inflammation by upregulating GSK-3β. Consequently, we established a podocyte-specific GSK-3β-KO mouse (GSK-3β^flox/flox, nphs2-cre^). Six-week-old GSK-3β-KO and control (CON; GSK-3β^flox/flox^) mice were injected intravenously with a podocyte-specific lncRNA Dlx6-os1 (exon 3) overexpression-lentivirus (Fig. 8a). As expected, GSK-3β expression reduced almost entirely in the GSK-3β-KO mice compared to the control mice (Fig. 8b), and the levels of lncRNA Dlx6-os1 increased in both groups compared to the non-injected control (CON; Fig. 8c). Next, we evaluated the uACR values in these mice. Notably, a significant elevation in the uACR values was observed in the flox/flox control mice 4 weeks after injection and in the GSK-3β-KO mice 5 weeks after injection (*P* < 0.05, Fig. 8d). Moreover, 5 weeks after injection, there was a clear increase in uACR between the control and KO mice (Fig. 8d). Next, we performed histological studies to compare the kidneys in the two models. PAS staining and EM indicated that the GSK-3β-KO mice had significantly improved morphology than the control mice (*P* < 0.05, Fig. 8e), as demonstrated by the foot process number and the GBM thickness, suggesting that GSK-3β could counteract the effects of Dlx6-os1 overexpression. In addition, we also studied the expression of podocin and Dlx6-os1 in the glomeruli of these mice. The FISH results confirmed the expression and localization of lncRNA Dlx6-os1, and the FISH immunofluorescence staining showed disrupted podocin expression in the glomeruli of the control mice with Dlx6-os1 overexpression (Fig. 8f). We also analyzed the expression levels of several podocyte markers using WB (Fig. 8g, h) and immunofluorescence staining (Fig. 8i–k), and we found a reduced expression of inflammatory and injury-associated factors in the GSK-3β-KO mice with podocyte-specific lncRNA Dlx6-os1 overexpression, compared to the control (Fig. 8i–k). The intensity of the immunofluorescence staining were analyzed and shown in Supplementary Fig. 9.

**Fig. 9:**
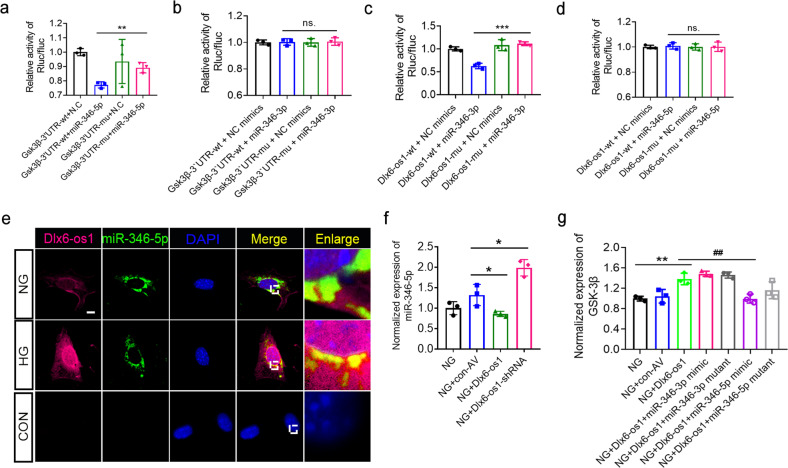
The regulatory effects of lncRNA Dlx6-os1 on the expression of GSK-3β through miR-346. **a** Luciferase reporter gene expression confirming that wild-type (WT) Gsk-3β 3’UTR can bind miR-346-5p. Mu, Mutant. **P < 0.01, one-way ANOVA plus Tukey’s multiple comparisons test; *n* = 3. **b** Luciferase reporter gene expression confirming that wild-type (WT) Gsk-3β 3’UTR do not bind miR-346-3p. Mu Mutant, ns no significant difference. One-way ANOVA plus Tukey’s multiple comparisons test; *n* = 3. **c** Luciferase reporter gene testing confirming that the wild-type (WT) lncRNA Dlx6-os1 can directly bind miR-346-3p. Mu, Mutant. ***P < 0.001. One-way ANOVA plus Tukey’s multiple comparisons test; *n* = 3. **d** Luciferase reporter gene testing confirming that the wild-type (WT) lncRNA Dlx6-os1 cannot directly bind miR-346-5p, Mu Mutant, ns no significant difference (one-way ANOVA plus Tukey’s multiple comparisons test); *n* = 3. **e** FISH confirming the colocalization of lncRNA Dlx6-os1 and miR-346-5p in MPC5 podocytes under different glucose conditions. The lncRNA Dlx6-os1 expression level is increased, and the extent of their colocalization (yellow area) is increased in podocytes treated with high glucose (HG), while the expression of miR-346-5p was reduced. **f** qRT-PCR detection of the expression levels of miR-346-5p in MPC5 podocytes after lncRNA Dlx6-os1 overexpression (NG + Dlx6-os1) or knockdown (NG + Dlx6-os1 shRNA). NG normal glucose. *P < 0.05; n = 3. **g** qRT-*P*CR detection of the expression levels of GSK-3β in MPC5 podocytes overexpressing lncRNA Dlx6-os1 with miR-346 (5p or 3p) mimics or mutant. **P < 0.01; ##*P* < 0.01 (One-way ANOVA plus Dunnett’s multiple comparisons test); *n* = 3.

Some lncRNAs, called competitive endogenous RNAs (ceRNAs), can act as endogenous target mimics regulating gene expression by competing with miRNAs [41–43]. Therefore, we checked whether lncRNA Dlx6-os1 could regulate the GSK-3β pathway acting as a ceRNA. Previous studies have reported that the microRNA miR-346 can exert a protective effect in db/db mice [44]. Hence, bioinformatics analysis was performed to assess the possible linkage among lncRNA Dlx6-os1, miR-346, and GSK-3β mRNA-binding sites. The possible binding sites among lncRNA DLX6-AS1, miR-346, and GSK-3β mRNA in humans are shown in Supplementary Fig. 10. Using bioinformatics, we predicted that lncRNA Dlx6-os1 and GSK-3β could competitively bind to the same site of the two miR-346 transcripts (Supplementary Fig. 11a–d; green). We set up a dual-luciferase reporting assay to directly assess the binding of miR-346-3p and miR-346-5p (accession no. MI0000634; http://www.mirbase.org/) to GSK-3β mRNA and Dlx6-os1. The results indicated that miR-346-5p could directly bind to the wild-type GSK-3β mRNA 3’UTR sequences but not the mutated ones (*P* < 0.01, Fig. 9a). In contrast, miR-346-3p did not directly interact with the wild-type GSK-3β mRNA 3’UTR sequence (Fig. 9b), but it could bind to Dlx6-os1 (Fig. 9c). Although miR-346-5p did not directly bind to Dlx6-os1 (Fig. 9d), we found the colocalization of miR-346-5p and Dlx6-os1 under both normal and HG culture conditions in the cytoplasm of MPC5 cells using FISH and immunofluorescence staining (Fig. 9e). Notably, unlike Dlx6-os1, miR-346-5p was not found in the nucleus in HG conditions, suggesting that Dlx6-os1 might play a different modulatory role there. In addition, we found that Dlx6-os1 overexpression or knockdown could modulate the expression levels of miR-346-5p (Fig. 9f), suggesting the existence of a different regulatory mechanism between Dlx6-os1 and miR-346-5p.

In addition, we examined the effects of Dlx6-os1 overexpression and miR-346 mimics and mutants (Supplementary Fig. 12a–c) on the expression of GSK-3β mRNA (Fig. 9g). The results indicated that Dlx6-os1 overexpression in MPC5 cells (NG conditions) could upregulate GSK-3β expression (Fig. 9g, in green), and this effect was abolished by miR-346-5p mimic expression (Fig. 9g, in purple). The miR-346-5p mutant and miR-346-3p did not affect GSK-3β, consistent with the luciferase binding assays (Fig. 9a, b). Therefore, we hypothesize that Dlx6-os1 plays a role in modulating GSK-3β by indirect interaction with miR-346-5p. These data suggested that the lncRNA Dlx6-os1 could regulate GSK-3β expression together with miR-346.

## Discussion

There is increasing interest in elucidating the molecular mechanisms underlying the pathogenesis of diabetic nephropathy, including the role of lncRNAs in the prevention and treatment of diabetes [27, 45, 46]. The present study aimed to better understand the role of lncRNAs in the development of DN, mainly focusing on the effects and mechanisms of the action underlying lncRNA DLX6-AS1-induced podocyte injury. LncRNA DLX6-AS1 (Dlx6-os1 in mouse) was the first lncRNA found to be involved in organogenesis [47]. DLX6-AS1 regulates gene expression in a brain region that produces GABAergic interneurons during development [48, 49] and works in conjunction with DLX homeodomain proteins to increase the effectiveness of the Dlx5/6 enhancer element within neural stem cells [50]. However, the potential role of lncRNA DLX6-AS1 in the development and progression of diabetes or DN has not been reported or suggested previously. The present study represents the first undertaking in this critical field of diabetic research and made several interesting discoveries.

First, we found increased expression of DLX-AS1 in DN patients (Fig. 1i) and a positive correlation between DLX6-AS1 expression and uACR in the urine, suggesting a link between DLX6-AS1 and the progression of diabetic nephropathy (Fig. 1j). Similarly, we found increased levels of Dlx6-os1 expression in diabetic mice compared with the non-diabetic controls (Fig. 2a, b) and a positive correlation between Dlx6-os1 expression and uACR of db/db mice (Fig. 2c). Interestingly, increased Dlx6-os1 expression levels were accompanied by a decrease in podocyte markers, including podocin, WT-1, and SYNPO, and an increase in inflammatory factors, such as IL-17 (Figs. 3g–l, 4g–l), suggesting a link between Dlx6-os1 and podocyte fitness in vivo. The mechanism of reduction in the podocyte number (reduced expression of WT-1) in db/m mice in the case of lncRNA Dlx6-os1 overexpression needs further exploration in the future. To further explore the effects of the lncRNA Dlx6-os1 in DN, we first set up the podocyte-specific Dlx6-os1-knockout mice under low-dose STZ treatment (Fig. 5a–f). We found specific-knockout Dlx6-os1 in podocytes alleviated albuminuria, podocyte injury (Fig. 5g–l), and inflammation (Fig. 5m–q) without changing blood glucose in mice with STZ treatment, which illustrated the crucial role of Dlx6-os1 in DN. However, IL-17 and B7-1 expressions in the glomeruli were not limited to the podocytes; hence, further studies are needed to address the role of other cell types in the DN progression [51]. We also determined the expression levels of lncRNA Dlx6-os1, inflammatory factors, and podocyte-specific marker proteins in cultured podocytes (MPC5 cell line) and observed the effects of HG (Fig. 6). Following exposure to HG concentration, the levels of lncRNA Dlx6-os1, claudin-1, and inflammatory factors increased, while podocyte marker proteins decreased (Fig. 6c–f). Further, our experiments with lncRNA Dlx6-os1 overexpression and shRNA-mediated knockdown demonstrated the role of lncRNA Dlx6-os1 in HG-induced inflammation and cellular damage in podocytes (Fig. 6g–m).

Further, an RNA-seq study was performed to explore the mechanisms by which lncRNA Dlx6-os1 caused podocyte injury (Fig. 7a–e), and several differentially expressed mRNAs were identified. Based on these results (Fig. 7f, g) and the previous literature [18, 20, 35], we selected GSK-3β as a possible target. Interestingly, Dlx6-os1 overexpression or knockdown led to increased (Fig. 7h, j, l, n) or decreased (Fig. 7i, k, m, o) levels of GSK-3β respectively, suggesting that Dlx6-os1 could modulate GSK-3β levels in MPC5 cells and primary podocytes isolated from mouse kidney. In addition, we created a podocyte-specific GSK-3β KO mouse and investigated the relationship between GSK-3β and podocyte damage, and we found that GSK-3β KO seemed to have a protective effect, as measured by uACR levels, histology, and WB (Fig. 8).

Finally, we found that the primary mechanism of action of Dlx6-os1 is through the regulation of the GSK-3β pathway via miR-346 (Fig. 9). We set up a dual-luciferase reporting assay using non-mutated and mutated sequences of Dlx6-os1 and GSK-3β and found that miR-346-5p could directly bind to the wild-type GSK-3β mRNA 3’UTR sequences, but not the mutated ones (Fig. 9a). Moreover, we detected colocalization of Dlx6-os1 and miR-346-5p in the cytoplasm (Fig. 9e), despite no direct interaction (Fig. 9d). In addition, we examined the effects of miR-346 mimics and mutant on the expression of GSK-3β mRNA. The results indicated that, in MPC5 cells, Dlx6-os1 overexpression (NG conditions) could upregulate GSK-3β expression, and this effect could be rescued by miR-346-5p mimic expression (Fig. 9g). However, miR-346-3p did not have a similar role. Our results suggest that miR-346-5p plays a role in the regulation of GSK-3β mRNA, despite the lack of direct interaction between Dlx6-os1 and miR-346-5p. Studies using FISH and immunofluorescence indicated the localization of lncRNA Dlx6-os1, but not miR-346-5p, in the nucleus under HG conditions (Fig. 9e), suggesting that Dlx6-os1 might play a different modulatory role there.

Interactions between lncRNA DLX6-AS1 (Dlx6-os1) and some miRNAs have been previously reported, although most studies were carried out in cancer models [52–55]. For instance, lncRNA DLX6-AS1 enhances osteosarcoma stemness via the regulation of miR-129-5p/DLK1 [56], promotes breast cancer progression via the regulation of the miR-505-3p/RUNX2 axis [57], cervical cancer progression by targeting the miR-16-5p/ARPP19 axis [58], and squamous cell carcinoma growth and invasion through the regulation of miR-376c [59]. It has also been reported that lncRNA DLX6-AS1 modulates glucose metabolism and the growth of gastric cancer cells by targeting miR-4290 [60]. Other reported molecular targets of lncRNA DLX6-AS1 include the PI3K/AKT/mTOR pathway [61], and the MMP-2 pathway [22]. However, the role of lncRNA DLX6-AS1 in diabetes has not been reported. Here, we show that Dlx6-os1 regulates GSK-3β by indirectly binding miR-346-5p, which is different from the traditional ceRNA mechanism where the lncRNA and the miR directly bind to each other. Our results provide the first evidence supporting the idea that lncRNA DLX6-AS1 (Dlx6-os1) plays a key role in the development and progression of DN. Although GSK-3β has been reported to have a major role in glomeruli podocyte injury [18–20, 62], our study is the first report demonstrating the involvement of the lncRNA DLX6-AS1-miR-346-GSK-3β pathway in the pathogenesis of DN.

**Fig. 10:**
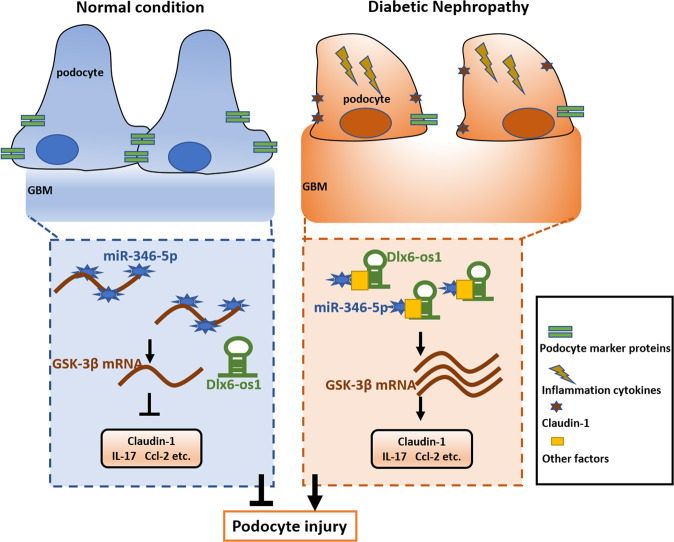
Proposed mechanism by which lncRNA Dlx6-os1 induces podocyte injury leading to diabetic neuropathy (DN). Left panel: Under normal circumstances, lncRNA Dlx6-os1 expression in renal podocytes is at a basal level, with no change or a slight reduction in diabetic individuals. Podocyte marker protein expression is normal, and inflammatory and injury-associated factors are rarely expressed. Right panel: In individuals with diabetic nephropathy, lncRNA Dlx6-os1 expression is increased, resulting in GSK-3β upregulation, thus leading to increased expression of inflammatory factors and cell damage-associated factors, podocyte injury and albuminuria.

Based on our experimental data, we concluded that lncRNA Dlx6-os1 directly damages podocytes. We herein propose a working model: as shown in Fig. 10, under normal circumstances, lncRNA Dlx6-os1 expression in renal podocytes is at a basal level, with no change or a slight reduction induced by diabetes. Podocyte marker protein expression is normal, and inflammatory and injury-associated factors are rarely expressed. However, in patients with diabetic nephropathy, lncRNA Dlx6-os1 expression increases, resulting in GSK-3β upregulation, thus leading to increased expression of inflammatory factors and cell damage-associated factors, podocyte injury, and albuminuria. These results are highly significant and provide new clues for developing targeted therapy or prevention strategies for DN. Future studies should explore the value of lncRNA DLX6-AS1 as a diagnostic marker and therapeutic target in preclinical and clinical settings. Our newly developed lncRNA Dlx6-os1 conditional or podocyte-specific knockout mice models are expected to be helpful in these types of studies for developing targeted therapies. Altogether, the data above suggested that DLX6-AS1 could play a role in the development and progression of podocyte injury and albuminuria in DN.

Besides DLX6-AS1, there are also several other lncRNAs with significant expression changes. For example, lncRNA DNAJC19 and MYLK-AS1 are significantly decreased in DN patients. DNAJC19 is closely related to the function of mitochondria [62, 63]. The downregulation of DNAJC19 is closely with mitochondrial injury-related cardiomyopathy, autism, and other diseases [64–67]. However, the role of lncRNA DNAJC19 in nephropathy is not clear. Because the kidney is also a mitochondria-rich organ, similar to the heart, we believe that the downregulation of DNAJC19 expression will also lead to kidney injury. lncRNA MYLK-AS1 is generally considered to be a tumor-promoting factor, and its decreased expression could inhibit tumorigenesis, such as nephroblastoma, gastric cancer, and hepatocellular carcinoma, through different mechanisms [19, 68–70]. On the other hand, our results showed that lncRNA RUNX1-IT1 was significantly upregulated in DN patients, similar to DLX6-AS1. Current studies showed that RUNX1-IT1 usually acts as a tumor suppressor in acute myeloid leukemia, colorectal cancer, and ovarian cancer [71–74]. At present, there are rare publications about those lncRNAs in kidney diseases, which are worth exploring further.

RNA-based therapeutics may represent a promising strategy for personalized and precision treatment of human diseases in the ascendant. According to a patient’s genetic profile, the activity of specific protective or pathogenic lncRNAs may be manipulated by introducing respective gain- and loss-of-function mutants. The unique tissue-specific characteristics of lncRNAs may be used to develop certain lncRNAs into the next-generation biomarkers for early diagnosis/prognosis of diabetes and DN. However, a number of challenges still need to be addressed to achieve these goals. But it is promising and feasible in the future thanks to the latest breakthroughs in the biotechnology of gene therapy [24, 75, 76].

## Supplementary information

Supplementary figures and legend

Supplementary methods

Original Data File

AJ-checklist

## Data Availability

Correspondence and requests for materials should be addressed to Prof. Zhangsuo Liu and Jia Guo. The results of RNA-seq information are available at https://www.ncbi.nlm.nih.gov/geo/query/acc.cgi?acc=GSE165241.
