# Supplementary material for: Long non-coding RNA DLX6-AS1 is the key mediator of glomerular podocyte injury and albuminuria in diabetic nephropathy by targeting the miR-346/GSK-3β signaling pathway
Source: Cell Death Dis. 2023 Feb 28;14(2):172. doi: 10.1038/s41419-023-05695-2 (PMC9975222; doi:10.1038/s41419-023-05695-2)
Supplement: Supplementary file 1 — Supplementary figures and legend [file 41419_2023_5695_MOESM1_ESM.docx]

**Supplementary Figures**

**
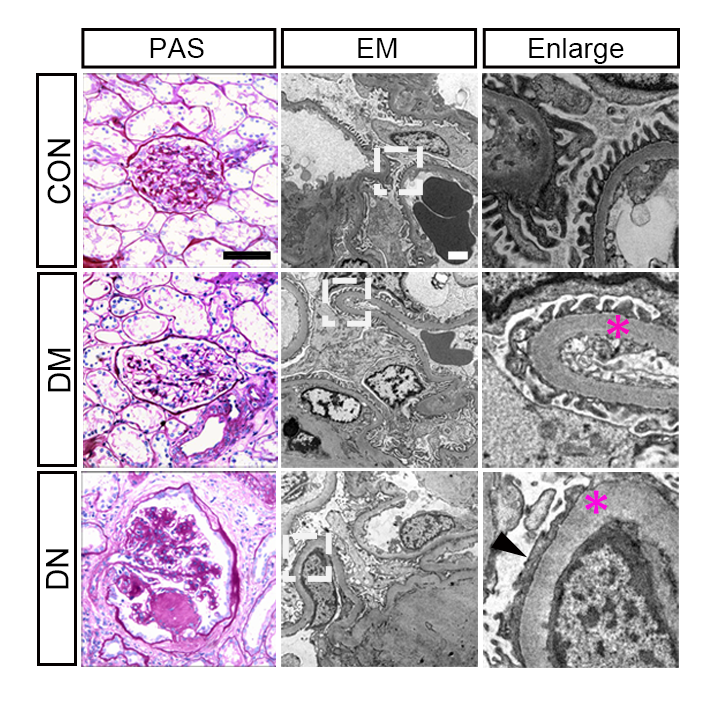
**

**Supplementary Fig. 1. Periodic Acid-Schiff (PAS) staining and electron microscopy (EM) to observe the structural changes in the kidney tissues and podocytes of diabetic nephropathy (DN) patients.** DN kidneys show increased matrix components, basement membrane thickening, and partial hardening using PAS staining. EM reveals significant thickening of the basement membrane (magenta asterisk) and foot process effacement (black arrow) in DN kidneys. PAS size bar = 50 µm; EM bar = 2 µm.


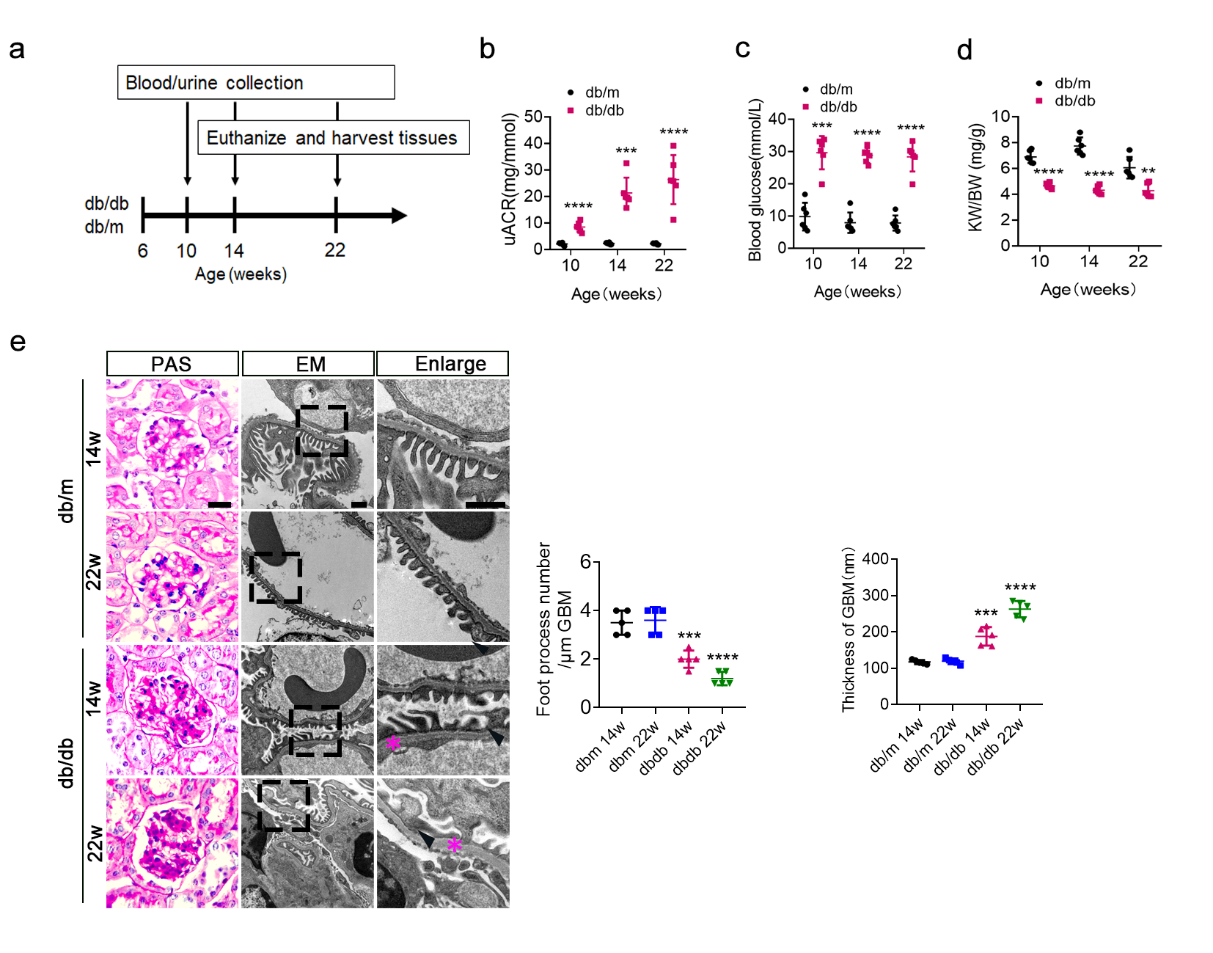


**Supplementary Fig. 2. Characteristics of db/db mice and db/m mice. a** The experimental flow chart. The animals were sacrificed at the age of 14 or 22 weeks. Blood, urine, and kidney tissue samples were collected. **b** Comparison of the urinary albumin/creatinine ratio (uACR) values in the db/db and db/m mice. ****P* < 0.001 vs. db/m; *****P* < 0.0001 vs. db/m (unpaired t-test). n = 6 mice/group. **c** Comparison of the random blood glucose levels in the db/db mice and db/m mice. ****P* < 0.001 vs. db/m; *****P* < 0.0001 vs. db/m (unpaired t-test). n = 6 mice/group. **d** Comparison of the kidney weight/body weight (KW/BW) ratios in the db/db mice and db/m mice. ***P* < 0.01 vs. db/m; *****P* < 0.0001 vs. db/m (unpaired t-test). n = 6 mice/group. **e** Periodic acid-Schiff (PAS) staining and electron microscopy (EM) analyses of the kidney tissues and podocytes from db/db mice and db/m mice at 14 and 22 weeks of age. PAS staining shows increased renal matrix deposition and thickened basement membranes in the db/db mice; EM reveals significantly increased basement membrane thickening (magenta asterisk) and podocyte foot process effacement (black arrow) in the db/db mice. Three observation fields per sample and n = 5 mice/group. PAS bar = 20 µm; EM bar = 1 µm.


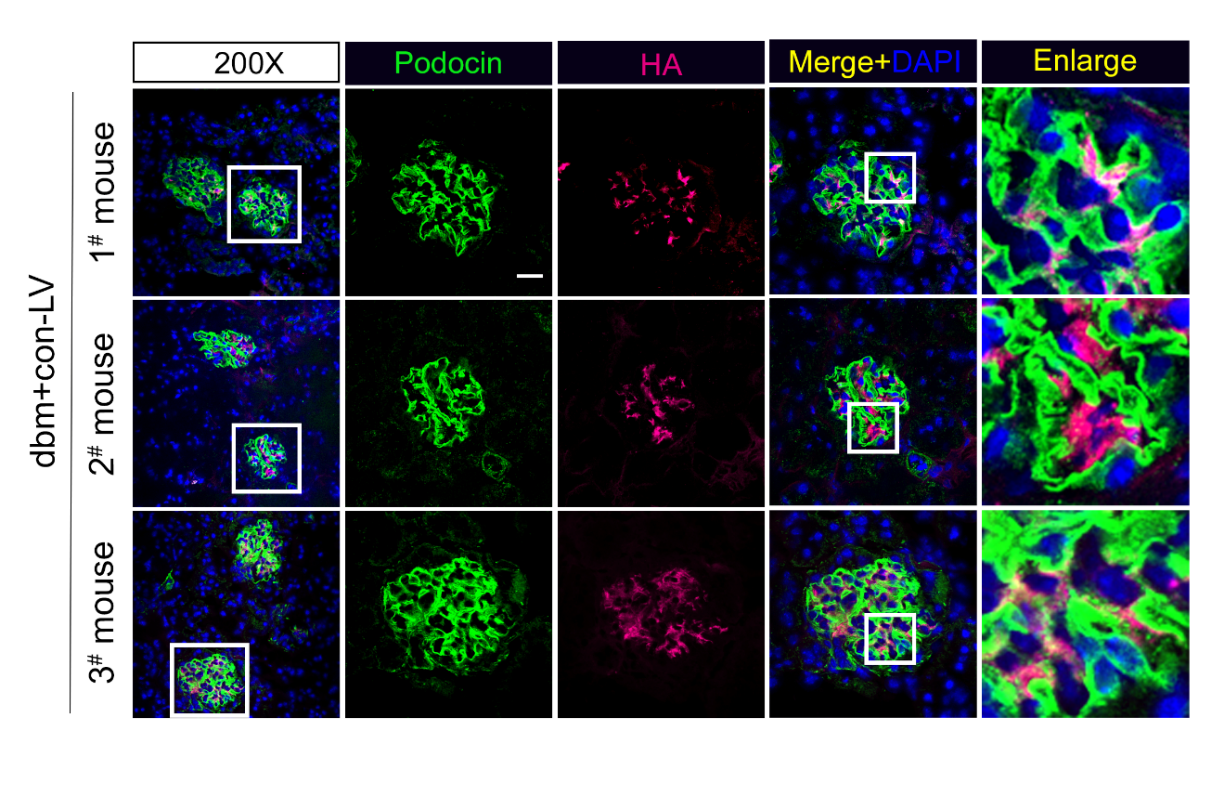


**Supplementary Fig. 3 Immunofluorescence staining showing the expression of HA-tag and podocyte marker protein podocin (expressed in the membrane) in the OCT-embedded frozen kidney tissue of mice.** The control viral vector used in this experiment is HA-tagged. Scale bar = 20 µm. (anti-HA antibody: M1008-1, HUABIO, China).


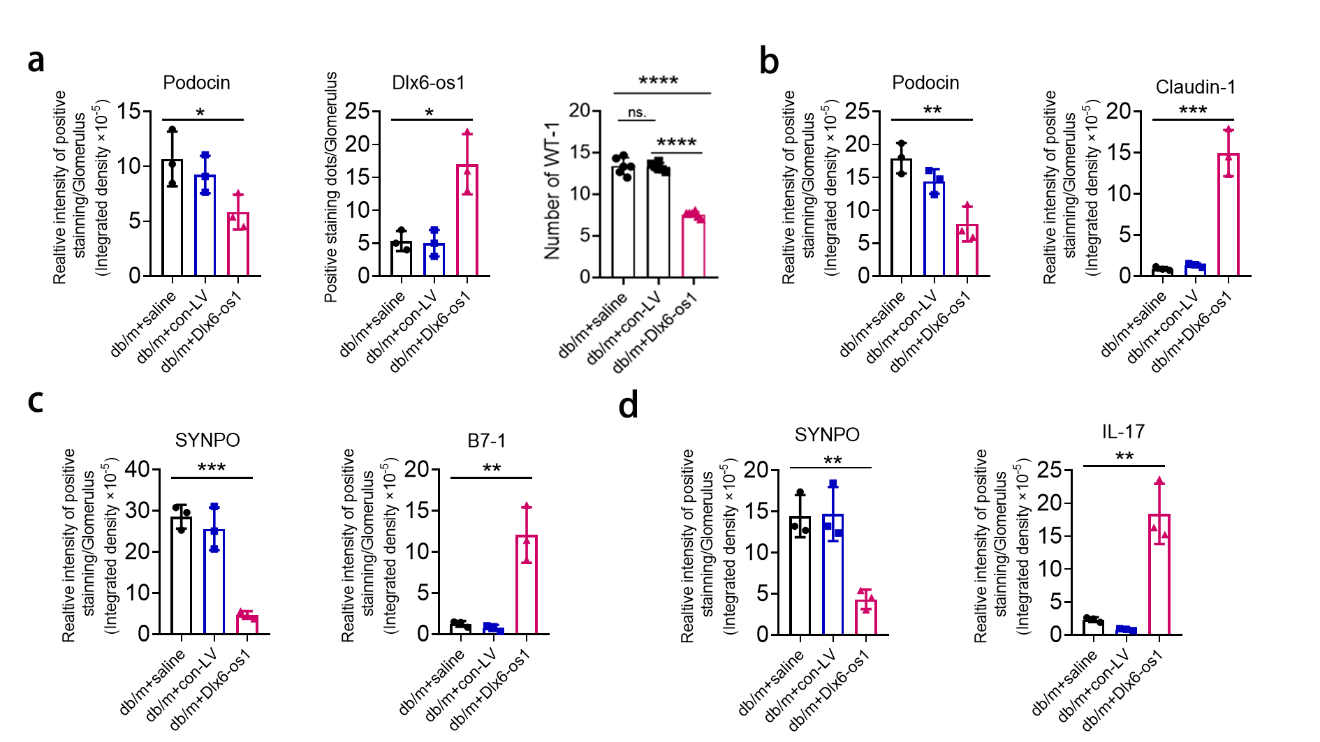


**Supplementary Fig. 4**  The intensity of the immunofluorescence staining shown in Fig.3 is quantified via ImageJ software and compared in the different samples. **a** The intensity analysis of Fig.3f. **b-d** The intensity analysis of Fig.3j-l. n = 3. **P* < 0.05, ***P* < 0.01, ****P* < 0.001, ns. NO significance (Unpaired t-test).


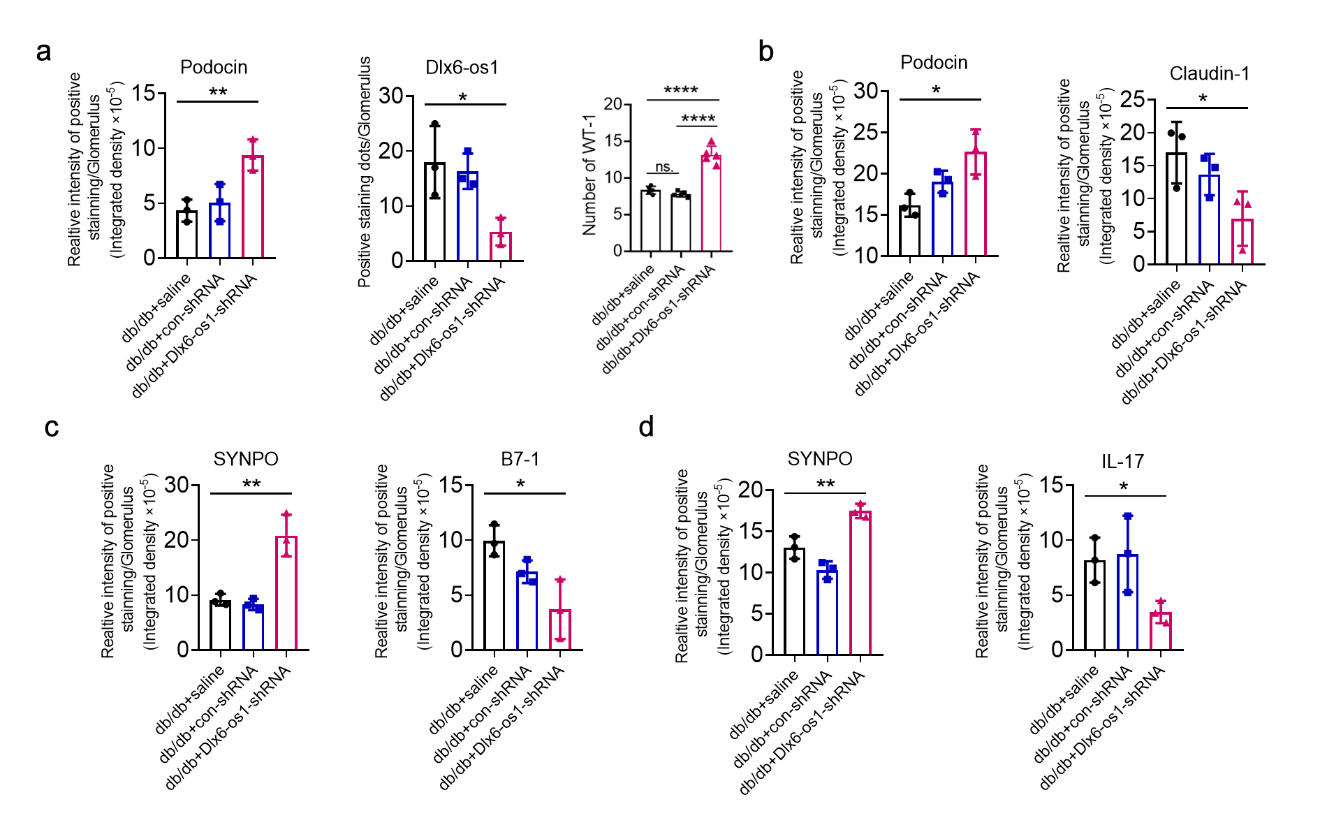


**Supplementary Fig. 5** The intensity of the immunofluorescence staining shown in Fig.4 is quantified via ImageJ software and compared in the different samples. **a** The intensity analysis of Fig.4g. **b-d** The intensity analysis of Fig.4j-l. n = 3. **P* < 0.05, ***P* < 0.01, *****P* < 0.0001, (Unpaired t-test).


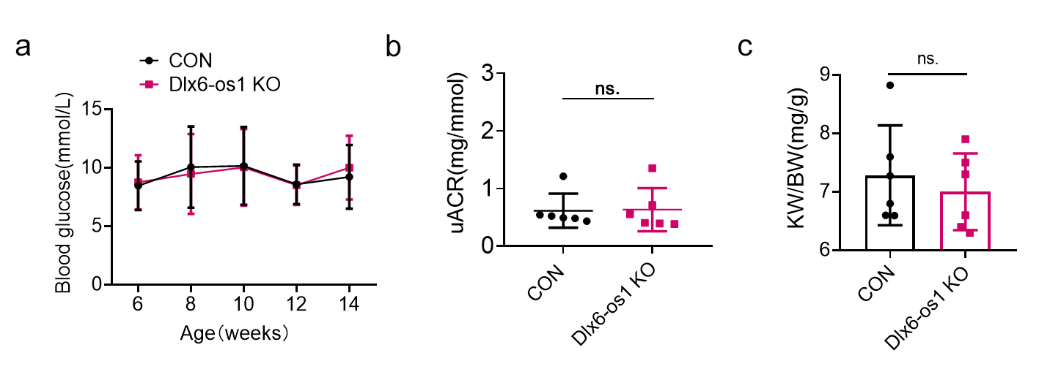


**Supplementary Fig. 6. Comparison of random blood glucose (RBG), urinary albumin/creatinine ratio (uACR), or kidney weight/body weight (KW/BW) ratio between the Dlx6-os1-KO and control mice.** No significant differences in the RBG **a**, uACR **b**, or KW/BW **c** between the control and Dlx6-os1 KO mice are observed. n = 6 mice/group. ns., no significant difference (unpaired t-test).


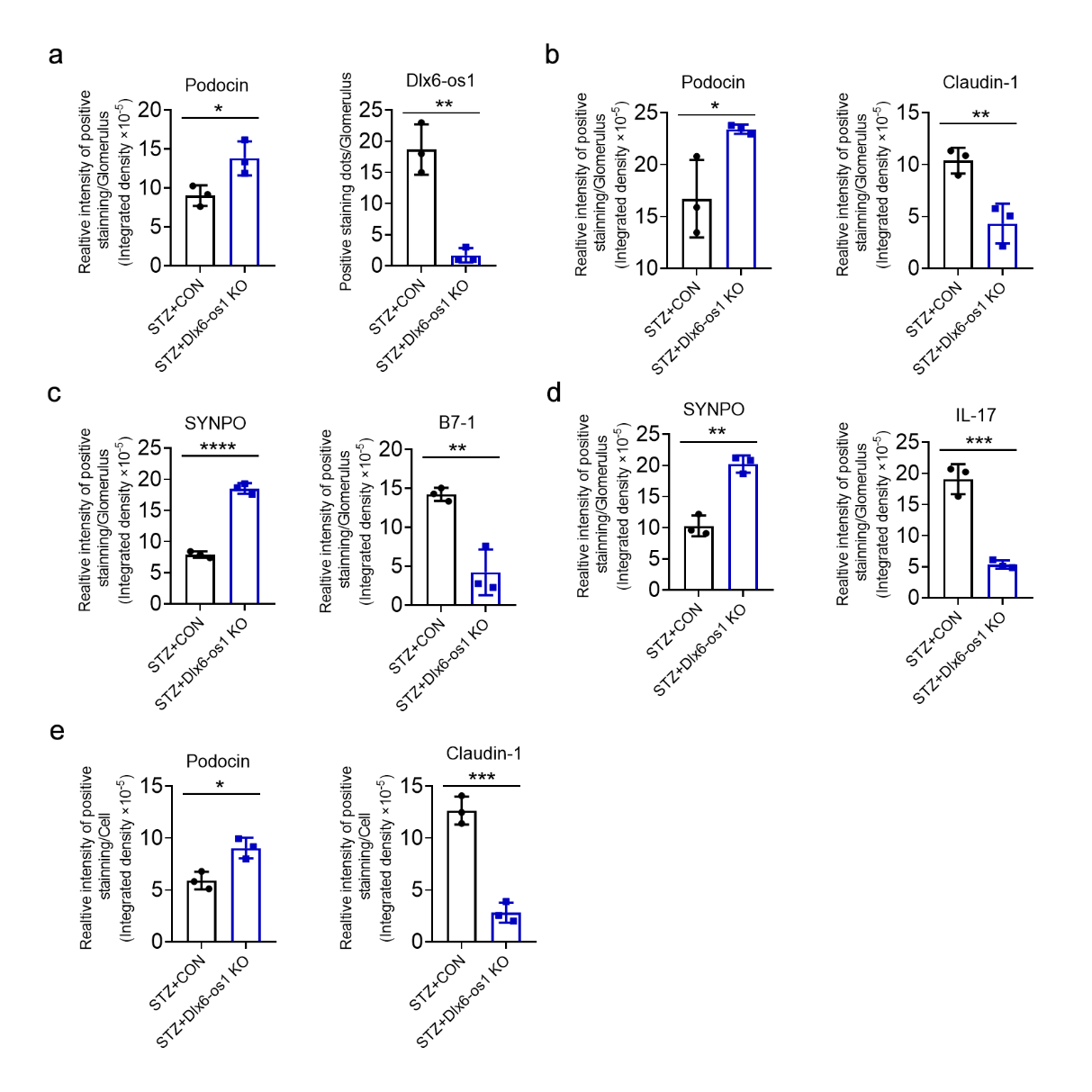


**Supplementary Fig. 7** The intensity of the immunofluorescence staining shown in Fig.5 is quantified via ImageJ software and compared in the different samples. **a** The intensity analysis of Fig.5k. **b-d** The intensity analysis of Fig.5m-o. **e** The intensity analysis of Fig.5q. n = 3. **P* < 0.05, ***P* < 0.01, ****P* < 0.001, *****P* < 0.0001 (Unpaired t-test).


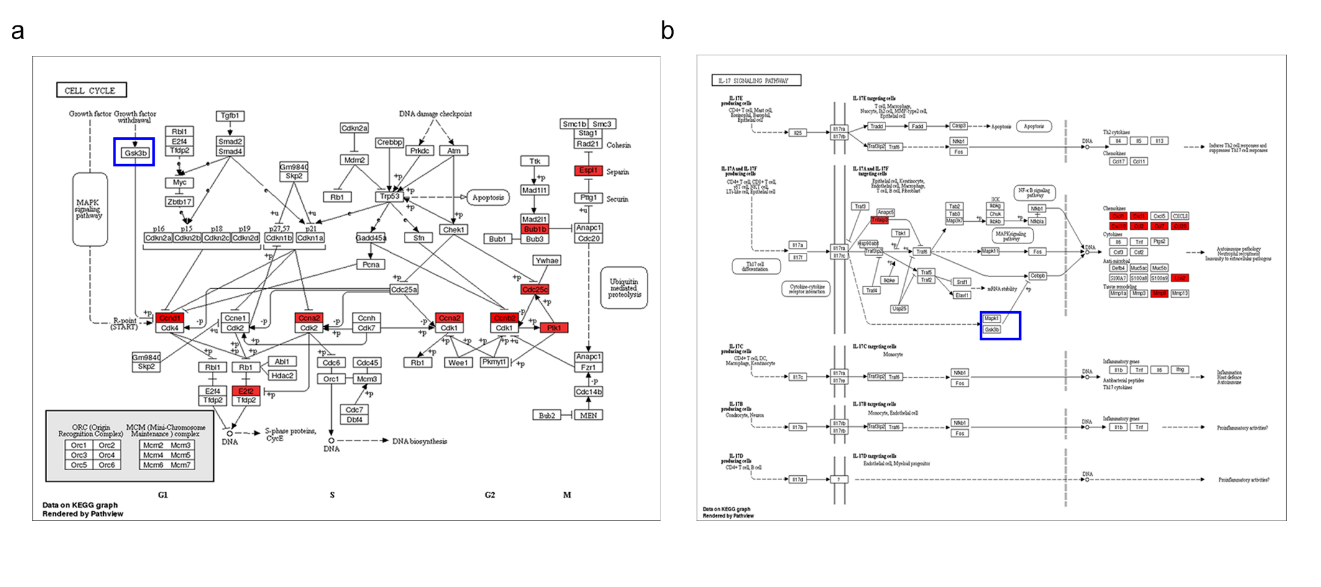


**Supplementary Fig. 8. The results of a Kyoto Encyclopedia of Genes and Genomes (KEGG) analysis. a** KEGG analyses of the cell cycle (mmu04110) involving GSK-3β (blue box) and **b** the IL-17 signaling pathway (mmu04657) involving GSK-3β (blue box).


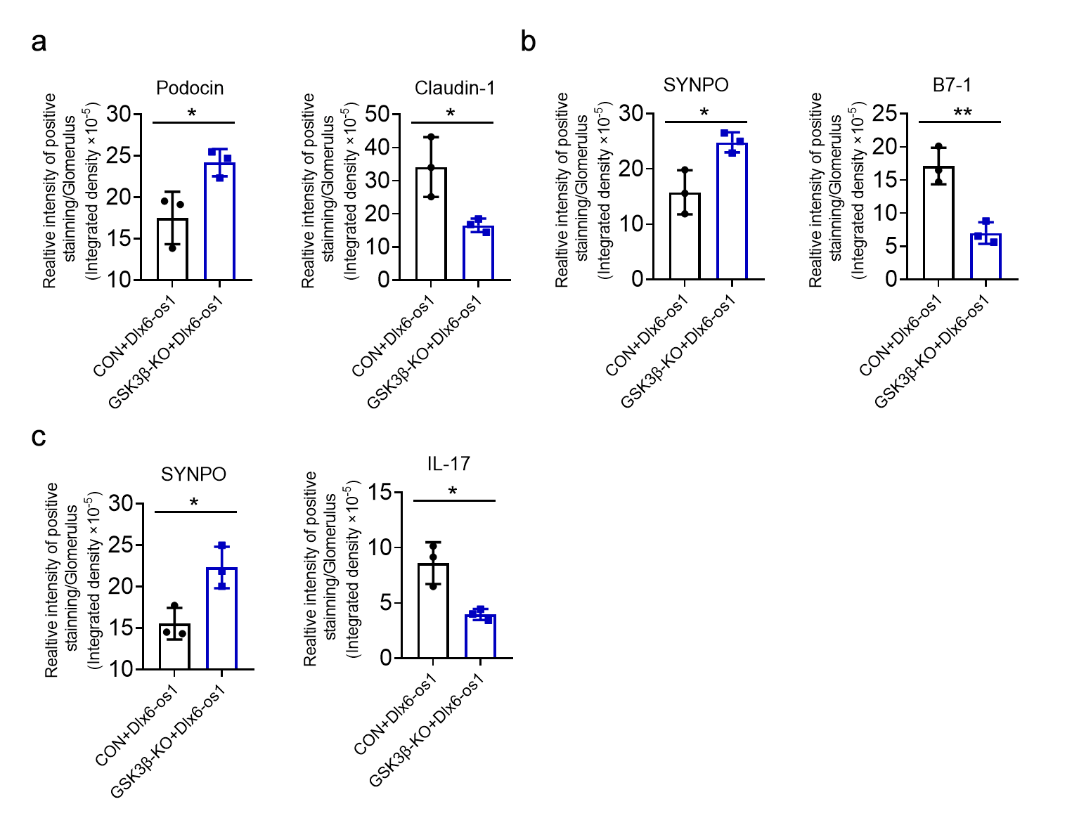


**Supplementary Fig. 9.** The intensity of the immunofluorescence staining shown in Fig.8 is quantified via ImageJ software and compared in the different samples. **a-c** The intensity analysis of Fig.8i-k. n = 3. *P < 0.05, ***P* < 0.01 (Unpaired t-test).


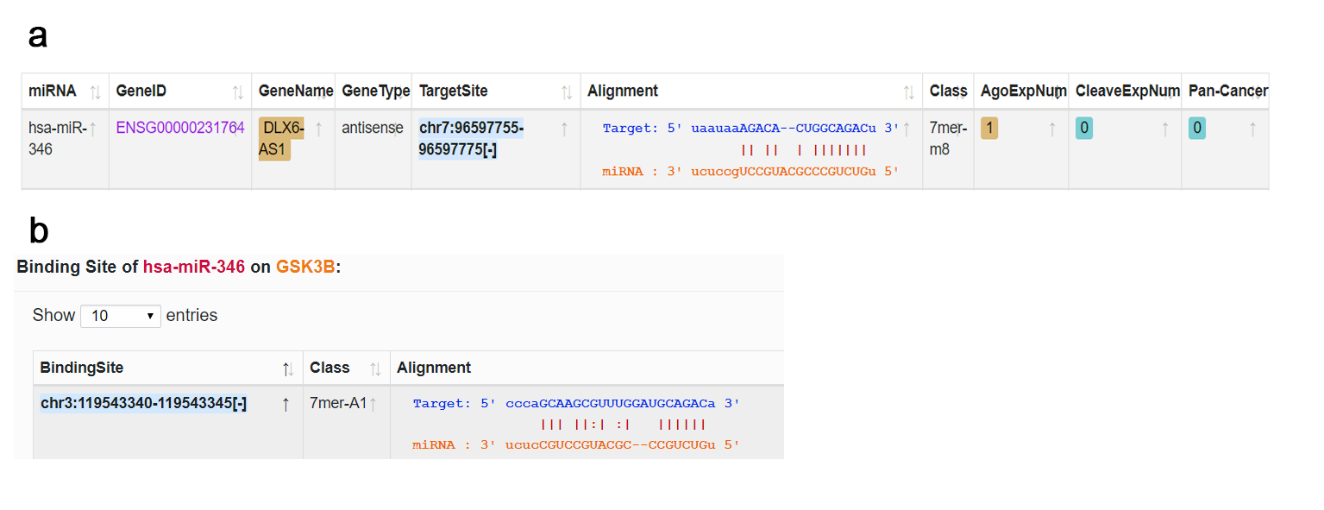


**Supplementary Fig. 10. The predicted binding sites** **determined** **using StarBase v2.0. (**[**http://starbase.sysu.edu.cn/**](http://starbase.sysu.edu.cn/)**, Guangzhou, China).** The predicted binding sites of **a** lncRNA Dlx6-os1 and **b** GSK**-**3β and miR-346 in humans.


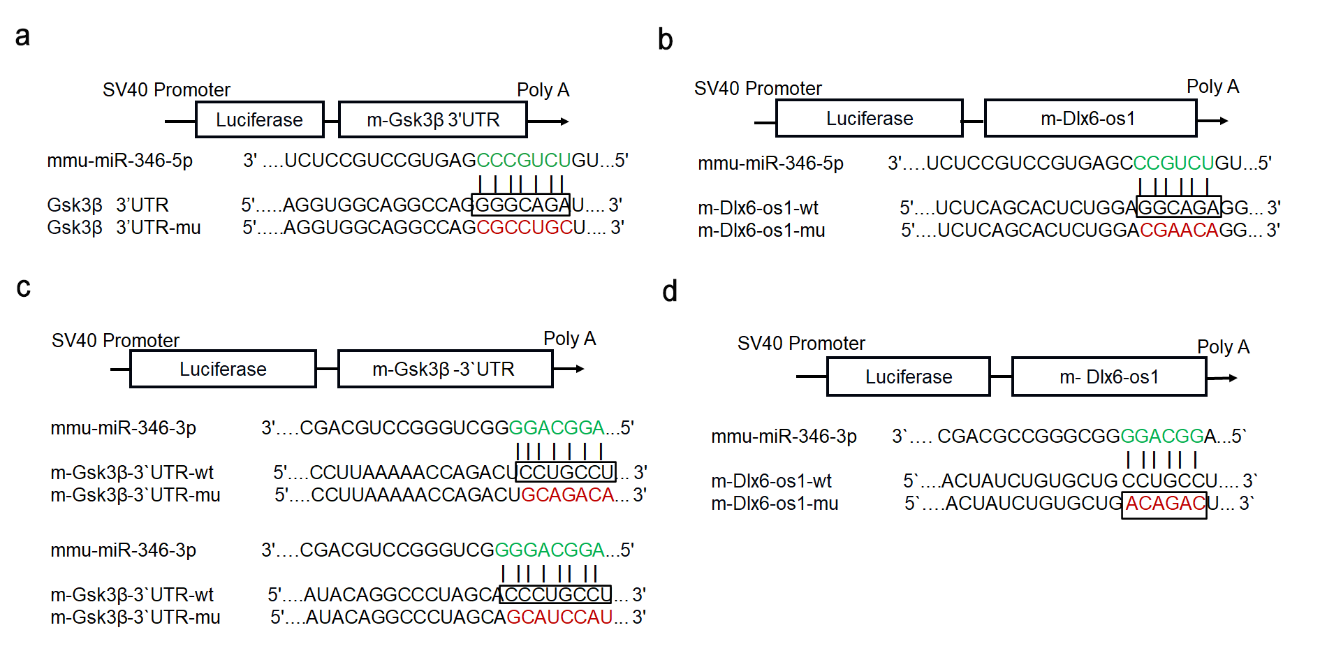


**Supplementary Fig. 11. The predicted binding sites determined using** **Sangon Biotech. Company (Shanghai, China).** The predicted binding sites of **a** GSK-3β and miR-346-5p, **b** lncRNA Dlx6-os1 and miR-346-5p, **c** GSK-3β and miR-346-3p, and **d** lncRNA Dlx6-os1 and miR-346-3p.


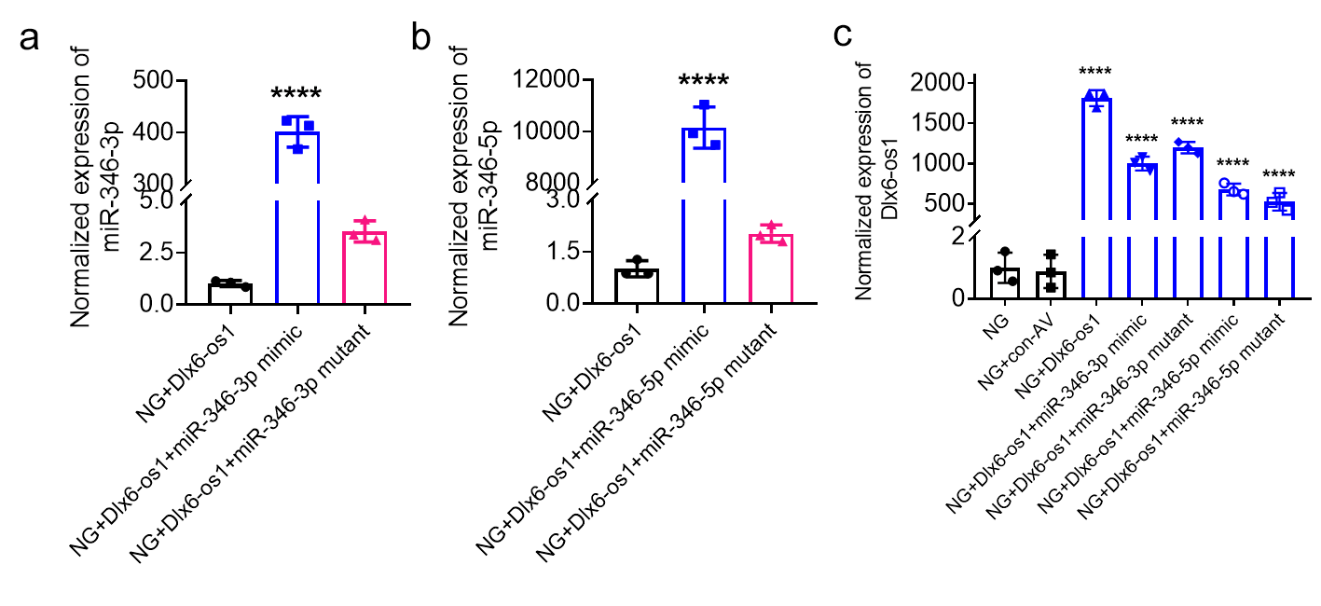


**Supplementary Fig. 12. The effects of Dlx6-os1 overexpression and miR-346 mimics and mutants. a** Changes in miR-346-3p expression after transfection of miR-346-3p mimics or mutant into MPC5 cells cultured under normal glucose (NG) condition with Dlx6-os1 overexpression. *****P* < 0.0001 vs. (NG+Dlx6-os1) (n = 3, one-way ANOVA plus Dunnett's multiple comparisons test). **b** Changes in miR-346-5p expression after transfection of miR-346-5p mimics or mutant into cells cultured under NG condition with Dlx6-os1 overexpression. *****P* < 0.0001 vs. (NG+Dlx6-os1) (n = 3, one-way ANOVA plus Dunnett's multiple comparisons test). **c** qRT-PCR detection of the expression levels of Dlx6-os1 in MPC5 podocytes overexpressing lncRNA Dlx6-os1 with or without miR-346 mimics or mutants. *****P* < 0.0001 vs. NG. (n = 3, one-way ANOVA plus Dunnett's multiple comparisons test).
