# Supplementary material for: Long non-coding RNA DLX6-AS1 is the key mediator of glomerular podocyte injury and albuminuria in diabetic nephropathy by targeting the miR-346/GSK-3β signaling pathway
Source: Cell Death Dis. 2023 Feb 28;14(2):172. doi: 10.1038/s41419-023-05695-2 (PMC9975222; doi:10.1038/s41419-023-05695-2)
Supplement: Supplementary file 2 — Supplementary methods [file 41419_2023_5695_MOESM2_ESM.docx]

***Supplementary Materials and Methods***

**Table 1. Characteristics of diabetic nephropathy (DN) and diabetes mellitus (DM) patients and controls**

| Parameters | CON  (n = 10) | DM  (n = 10) | DN  (n = 12) |
| --- | --- | --- | --- |
| Age (years) | 53.3 ± 10.75 | 53.7 ± 12.84 | 56.08 ± 8.70 |
| Duration of DM (years) | - | 9.64 ± 4.74 | 11.92 ± 6.43 |
| Men:women | 5:5 | 6:4 | 8:4 |
| SBP (mmHg) | 131.8 ± 17.57 | 122 ± 12.88 | 142 ± 20.26 |
| DBP (mmHg) | 81.7 ± 13.46 | 81.3 ± 9.53 | 87.5 ± 10.02 |
| ACR (mg/mmol) | 1.75 ± 2.64 | 0.69 ± 0.06 | 273.38 ± 127.76 |
| Hemoglobin (g/L) | 144.34 ± 16.38 | 131.86 ± 15.11 | 114.30 ± 22.13 |
| HbA1c (%) | - | 0.08 ± 0.02 | 0.07 ± 0.01 |
| Glucose (mmol/L) | 5.32 ± 0.51 | 6.98 ± 1.58 | 6.30 ± 2.18 |
| BUN (mmol/L) | 4.46 ± 0.84 | 5.69 ± 1.99 | 9.95 ± 4.36 |
| Cr, μmol/L | 64.3 ± 15.23 | 68.7 ± 18.43 | 148.33 ± 99.62 |
| Uric acid (μmol/L) | 270.8 ± 73.34 | 311.7 ± 146.83 | 398.25 ± 105.49 |
| Total cholesterol (mmol/L) | 4.62 ± 0.64 | 4.44 ± 1.40 | 5.70 ± 1.76 |
| Triglyceride (mmol/L) | 1.36 ± 0.48 | 1.88 ± 0.82 | 1.99 ± 0.94 |
| LDL cholesterol (mmol/L) | 2.80 ± 0.48 | 2.88 ± 1.14 | 3.80 ± 1.44 |
| HDL cholesterol (mmol/L) | 1.41 ± 0.38 | 1.05 ± 0.27 | 1.30 ± 0.45 |
| eGFR (mL/min/1.73 m^2^) | 100.43 ± 13.42 | 97.52 ± 17.71 | 55.55 ± 27.54 |

**LncRNA chip assay.** The assay was completed with assistance from Aksomics Biotech. Co. Ltd, (Shanghai, China) using Arraystar human lncRNA chip V3.0 (Arraystar Inc. Rockville, MD, USA) to analyze various biological samples, including plasma, urine, and kidney tissues. Sample labeling and chip hybridization were performed according to the Agilent One-Color Microarray-Based Gene Expression Analysis protocol (Agilent Technology Inc. Santa Clara, USA). The hybrid chip was finally scanned using an Agilent DNA Microarray Scanner (part number G2505C). The raw data were analyzed following quantile standardization, using GeneSpring GX v12.1 software (Agilent Technologies). The differentially expressed lncRNAs or mRNAs between the two groups were screened based on the P-value/FDR (*P* < 0.05, fold change > 2.0).

**PCR reagents.** The RNA extraction kit was obtained from Qiagen (RNeasy Mini Kit, Cat. # 74104, Hilden, Germany). The lncRNA and mRNA reverse transcription kit was obtained from Thermo Fisher (Revert Aid First Strand cDNA Synthesis Kit, Cat. # K1622). The qPCR kit was also purchased from Thermo Fisher (Maxima SYBR Green qPCR Master Mix (2X), Shanghai, China), with a separate ROX vial (Cat. # K0251). The miRNA reverse transcription and qPCR kits were obtained from Shanghai Novland Co. Ltd. [Two Step Stemaim -it miR qRT -PCR Quantitation Kit (SYBR Green), Cat. # LM-0101A, China]. The details of the reaction mixture are listed in Table 2. Each experiment was repeated three times, and the average results were reported.

**Table 2. The PCR reaction mixture**

| Reagent | Volume | Final concentration |
| --- | --- | --- |
| Maxima SYBR Green qPCR Master Mix (2× ) | 12.5 μL | - |
| Forward Primer (10 μM) | 0.75 μL | 0.375 μM |
| Reverse Primer (10 μM) | 0.75 μL | 0.375 μM |
| Template cDNA | 2.0 μL | - |
| RNase-free water | 9.0 μL | - |
| Total volume | 25.0 μL | - |

The PCR reaction conditions are listed in Table 3.

**Table 3. PCR reaction conditions**

| Step | Temperature (℃) | Time (s) | Number of cycles | |
| --- | --- | --- | --- | --- |
| Pre-denaturation | 95 | 600 | | - |
| Denaturation | 95 | 15 | | 40 |
| Annealing | 60 | 30 | | 40 |
| Extension | 72 | 30 | | 40 |

**PCR primer sequences.** Primers for human lncRNA DLX6-AS1 and the internal reference (*GAPDH*) were synthesized by the Bioengineering (Shanghai, China) Corporation.

| Human lncRNA *DLX6* -*AS*1 (product size 122 bp) | |
| --- | --- |
| Upstream | CCACCCACTGAGAGAAGAGG |
| Downstream | CCTCCAAGCAATTGTCCAGT |
| Human *GAPDH* | |
| Upstream | CAGGAGGCATTGCTGATGAT |
| Downstream | GAAGGCTGGGGCTCATTT |

Primers for mouse LncRNA Dlx6-os1 and the internal reference (*GAPDH*) were as follows:

Mouse lncRNA Dlx6-os1 F: 5'- CACACGTGGAGTCAGCAAAT- 3'

R: 3'- CTCGGAGCTTTCAAACAAGG- 5'

Mouse lncRNA Dlx6-os1 （overexpression）F: 5'- GCTCCAGTGGCCAGTTTCAA- 3'

R: 3'-TGGGAATAAGGAAAGAACAGGAGGA -5'

Mouse GAPDH F: 5'- GGTTGTCTCCTGCGACTTCA- 3'

R: 3'- TGGTCCAGGGTTTCTTACTCC- 5'

Primers for mouse miR-346-5p, mutant and the internal reference were designed by Bo Rui Biotech. Co., Ltd. (Guangzhou, China), and the sequences were as follows:

miR-346-5p Primer-F: 5'-AATGTCTGCCCGAGTGCCT- 3'

                 miR-346-5p Primer-R: 3'-ATTCCATGTTGTCCACAGTCTCC-5'

mmu-miR-346-3p MUT 5`-ACCGUCCCGCGGGCCGCAGC- 3`

mmu-miR-346-5p MUT 5` -UCAGACGGCGAGUGCCUGCCUCU-3`

micrON mmu-miR-346-5p mimic #miR10000597-1-5

micrON mmu-miR-346-3p mimic #miR11272590046-1-5

micrOFF mmu-miR-346-5p inhibitor #miR20000597-1-5

micrOFF mmu-miR-346-3p inhibitor #miR21272590657-1-5

Bulge-loop mmu-miR-346-3p primer set, 200T #MQPS0002751-1-200

Bulge-loop U6 qPCR primer set, 200T #MQPS0000002-1-200

The primers for mouse Gsk-3β mRNA were as follows:

Gsk-3β Primer-F: 5'- GGCAGCAAGGTAACCACAGT- 3'

Gsk-3β Primer-R: 3'- GATGGCAACCAGTTCTCCAG- 5'

**Podocyte-specific knockout of lncRNA Dlx6-os1 in mice.** The mouse model was established with assistance from the Cyagen Biological Company (Suzhou, China). Systemic conditional knockout of lncRNA Dlx6-os1 was established in C57BL/6J mice (Dlx6-os1 flox /+). These mice were inbred to generate the Dlx6-os1 flox/flox mice, which were crossed with nphs2-Cre C57BL/6J mice (bearing the Cre recombinase and NPHS2 promoter fragment) to generate podocyte-specific lncRNA Dlx6-os1- KO mice (Dlx6-os1 flox/flox, nphs2-cre mice). Dlx6-os1-flox/+ mice could be directly crossed with the nphs2-cre mice to generate Dlx6-os1-flox /+, nphs2-Cre mice, which were then inbred to obtain Dlx6-os1-flox/flox, nphs2-Cre mice. Podocyte-specific knockout of lncRNA Dlx6-os1 was confirmed using a routine PCR protocol to genotype tail DNA samples with the following primer pairs:

1) **Neo PCR**:

F1: 5 ' -TGGGAGGCTTTTGAGTTAGGAAT-3’;

R1: 5 ' -ATTGGACCATCTTACTGGACAGG-3’;

Target products**:** Wild type (WT): 222 bp; Mutant (MT): 327 bp

2) **NPHS2-cre PCR:**

F2: 5 ' -CGGTTATTCAACTTGCACCA-3’

R2: 5 ' -GCGCTGCTGCTCCAG-3’

Target products**:** Wild type (WT): NA Mutant (MT): 200 bp

3) **5'arm PCR**

F3: 5 ' -TTGATGGTTTTGGTAGATTATGCCC-3’

R3: 5 ' -TGAAAAGTCAGAAGCACTGTTACC-3’

Target products: Wild type (WT): 170 bp; Mutant (MT): 257 bp

- The construction of knockout mice based on the information on the ensemble website.

http://asia.ensembl.org/Mus_musculus/Transcript/Summary?db=core;g=ENSMUSG00000090063;r=6:6820543-6871592;t=ENSMUST00000159568.


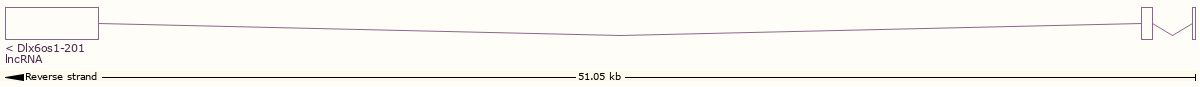


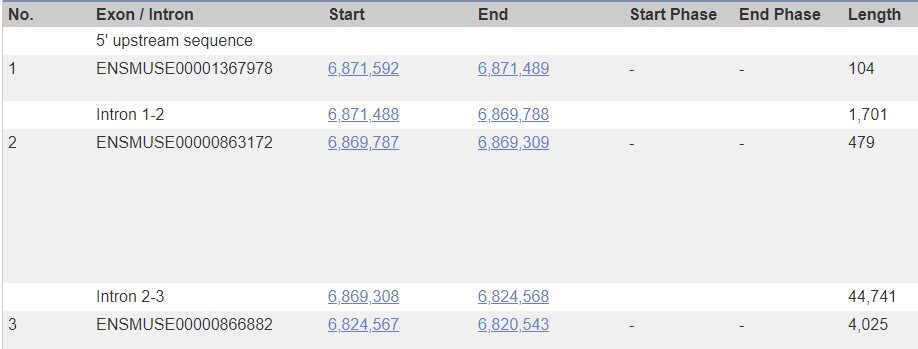


Mouse genomic fragments containing homology arms (HAs) and conditional knockout (cKO) region were amplified from BAC clone by using high fidelity Taq DNA polymerase, and were sequentially assembled into a targeting vector together with recombination sites and selection markers shown below.


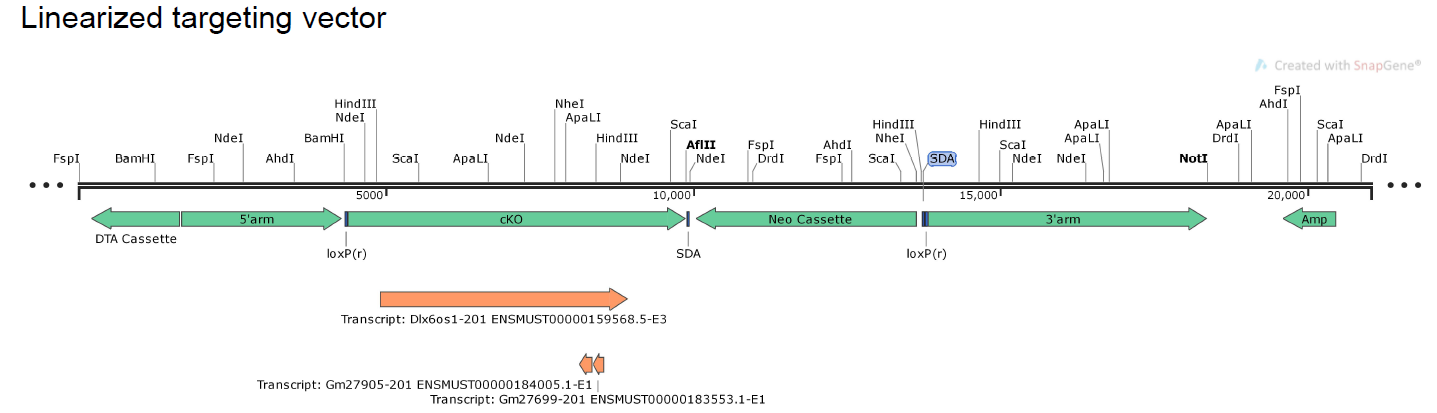


**The primers used for testing the knockout of exon 3 in primary isolated podocytes of Dlx6-os1 KO and control mice:**

mDlx6os1-E2-F: GGGAGCTACGAACCCAAAGT

mDlx6os1-E3-R: AGCAGTGGGAAAGCAATCCA 230bp

**Mouse lncRNA Dlx6-os1-shRNA adenovirus packaging fragment sequences****.** The adenovirus overexpressing lncRNA Dlx6-os1 (full-length) or specific knockdown shRNA and control adenovirus used in these experiments were designed and synthesized by Hanbio Co., Ltd. (Shanghai, China).

The sequences of the control (con-shRNA) siRNA and shRNA were as follows:

siRNA sequence: TTCTCCGAACGTGTCACGTAA

shRNA sequence

Top strand: aattcGTTCTCCGAACGTGTCACGTAATTCAAGAGATTACGTGACACGTTCGGA

GAATTTTTTg

Bottom strand: gatccAAAAAATTCTCCGAACGTGTCACGTAATCTCTTGAATTACGTGACACGT

TCGGAGAACg

The lncRNA Dlx6-os1-shRNA sequences were as follows:

siRNA1: GAGATATTGGCAATGACACTCTTGT

shRNA1(SH1)

Top strand: aattcGAGATATTGGCAATGACACTCTTGTTTCAAGAGAACAAGAGTGTCAT

TGCCAATATCTCTTTTTTg

Bottom strand: gatccAAAAAAGAGATATTGGCAATGACACTCTTGTTCTCTTGAAACAAGAGT

GTCATTGCCAATATCTCg

siRNA2: CGAGGAATAGCTTCCTCCAGGTAAT

shRNA2(SH2)

Top strand: aattcGCGAGGAATAGCTTCCTCCAGGTAATTTCAAGAGAATTACCTGGAGG

AAGCTATTCCTCGTTTTTTg

Bottom strand: gatccAAAAAACGAGGAATAGCTTCCTCCAGGTAATTCTCTTGAAATTACCT

GGAGGAAGCTATTCCTCGCg

 siRNA3: CAGTGAATGCCTGTCTCACTTAACA

shRNA3(SH3)

Top strand: aattcGCAGTGAATGCCTGTCTCACTTAACATTCAAGAGATGTTAAGTGAGA

CAGGCATTCACTGTTTTTTg

Bottom strand: gatccAAAAAACAGTGAATGCCTGTCTCACTTAACATCTCTTGAATGTTAAGT

GAGACAGGCATTCACTGCg

The lentivirus used for the overexpression of lncRNA Dlx6-os1 and shRNA knockdown, as well as the control lentivirus used in these experiments, were obtained from Hanbio Co., Ltd. (Shanghai, China).

**Mouse lncRNA Dlx6-os1-shRNA** **lentivirus packaging fragment sequences:**

shRNA3(SH3)

Top strand: aattcGCAGTGAATGCCTGTCTCACTTAACATTCAAGAGATGTTAAGTGAGA

CAGGCATTCACTGTTTTTTg

Bottom strand: gatccAAAAAACAGTGAATGCCTGTCTCACTTAACATCTCTTGAATGTTAAGT

GAGACAGGCATTCACTGCg

**The sequence for the overexpression of lncRNA DLX6-os1 was as follows:**

GCTCCAGTGGCCAGTTTCAAAATACCCTCCCTTTTGATGTTAGGTTACATAAACATTGTTCTTTTTTAGGGAGGGTCTCTTTTATCAACTTTTAAAAACACACATCAGGTTCTCTGGTATTAAAAAGATGCCATCTCTGAGTCCCCTACTATCTGTGCTGCCTGCCTTTCCTCCTGTTCTTTCCTTATTCCCATCCCTATTGAACTTGTGCTATGCAGTATGCATCAGGTATGTGTTAGCTTTGGGGATACATGATAGATAAACTGGACACACAGGGTCTTCCCATTCTCTTCTGGAATTTTCTTTGGAGGGAGCCTCTTGTATCTAGACAGACCGTGCTGTGGTACCCCAGAGGTAACCACCTACAGGCTTCACTCTGCCTAAGCAATTTTGCTGTGCACTAAGATACACATTCAAGTAACTTTAGATTACCACAATAACTTTCTCCAGGTATGAGGAAAAGAGATAATTTACTTCTGAGATGTGTATAGGATAGCCCTCCATCCTGGGAAGAACAGTGACTACTCCCTGCATCCCGACCTTGCCCAGGGAAAGCTAATGTTTCTCTGTGTTATCCCTGTGACTTGCCACTTCTTTAAAAAGGAATGGGCAAACAATAAACAGACAAAAATGTTGTCTGACCTCATTGGAAATCCTTTTAAGAATTAATCCTTTCTATCTCCTTCATTATCAACAAATCTATTGAATACTTATCTCTGAGTCCAGGGCATATTTTATAATACATAAAACAATGGAATTTCAAAATTGGAGCACTGACATACAATATTGGTTTTGAGTATTTTTATTATAGGGAATGACTTTAGACATTGCAATTTATGACTTAACTGATAAAATGGATGACTCTTGACTTTCAATTTTCATTTTCAGTTCAGTCGAGGAATAGCTTCCTCCAGGTAATGTCTATACTTTCCTATGACTAAGGGCTCTAACTATCTCTGTTGCTTTTCTTTATGTAGGCATATGTTAGTATTTATTTTCTATATGACAAATGTATTAAAGAAAGCATGAAATTAATGAGATAAACTTTTCAGATAGGAGTTTAGAAAATCAAGGGGCCAAGATAAATAAATGAAAAATCAACTTAAATAATTAACATATTCCAGATATATTGGAATAAATGTTTATTGTACCCTTTTGGTTTTGTCTTGGGTTATTTTTTTCTTATCTCACTGATTTTTTTTTCTTTCCTTTTTAGCTTTTTTGTCTTTTTTGATTTTTGTTGTTGCGTTTCTCCTTTTTTTTTTTCTTGTTGATGTTGTTTGTTTGTTTGTTTGTTTGTTTTTTGAGAAAGAACAGAAGGTTGGTTGGATAGGGAGGTGGGGAAGATCTATCTGGATGGAGTTGGGAGGAGGGAAAATACACGATCAAAATATATTTTGTGATGGGCAGGGCATAGTGGTACATGTCTTTAATCTCAGCACTCTGGAGGCAGAGGCAGGTGGATCTCTATGAGATGGAGGCTAGCCTGATATACAAAGTGAGACCAGAACATAGGGCTGCCTCAAAAACTTTATATATATATTAAAAATGTTTGCTTTTTGAGACAGTCACAGATAACCAAAACTGATCTTGTAATGATGTAAACATGTCCAGCTAATTTTCAAATATTGTAGGGCAGCATTTCTCCCTTTGTGCACACGTGGAGTCAGCAAATCCATATAATTCTAACCATTCTGGTGAAAAGGAGAACACTCGGCCAAGCATCTCACACTTCCAAGTGTGAAGCCTTGTTTGAAAGCTCCGAG

**Vendors for procurement of antibodies used in the present study were as follows:**

| **Antibody** | **Vendor** |
| --- | --- |
| Mouse monoclonal Anti-B7-1 Antibody (F-7) (sc-376012)  Rabbit polyclonal Anti-Synaptopodin antibody (ab224491) | Santa Cruz  Abcam |
| Rabbit polyclonal Anti-IL-17A antibody (ab79056)  Mouse monoclonal Anti-Synaptopodin antibody (D-9, sc-515842) | Abcam  Santa Cruz |
|  |  |
| Mouse monoclonal Anti-Claudin 1 antibody (37-4900)  Rabbit monoclonal Recombinant Anti-NPHS2 antibody [EPR13820] (ab181143) | Invitrogen  Abcam |
| Mouse monoclonal anti-F-actin antibody (ab205) | Abcam |
| Rabbit GAPDH polyclonal antibody (AB-P-R001) | Hangzhou Xianzhi Biological Company |
| Mouse GSK-3β monoclonal [3D10] antibody (ab93926) | Abcam |
| Rabbit mAb GSK-3β (D5C5Z) XP® (12456) | Cell Signaling Technology |
