# Supplementary material for: Long non-coding RNA DLX6-AS1 is the key mediator of glomerular podocyte injury and albuminuria in diabetic nephropathy by targeting the miR-346/GSK-3β signaling pathway
Source: Cell Death Dis. 2023 Feb 28;14(2):172. doi: 10.1038/s41419-023-05695-2 (PMC9975222; doi:10.1038/s41419-023-05695-2)

Figure2d

Podocin claudin-1


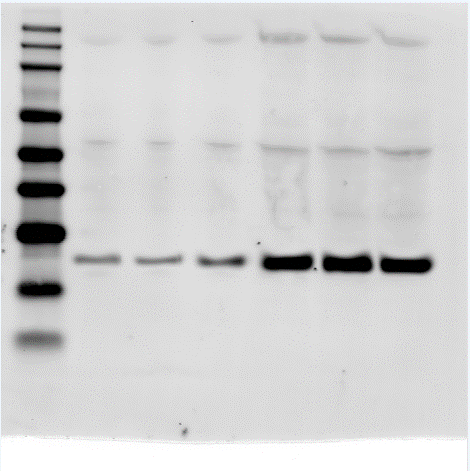

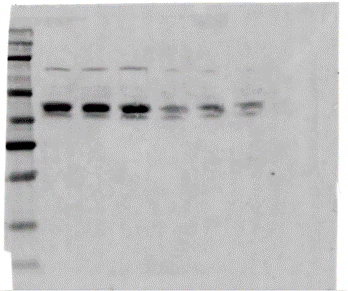


B7-1 IL-17


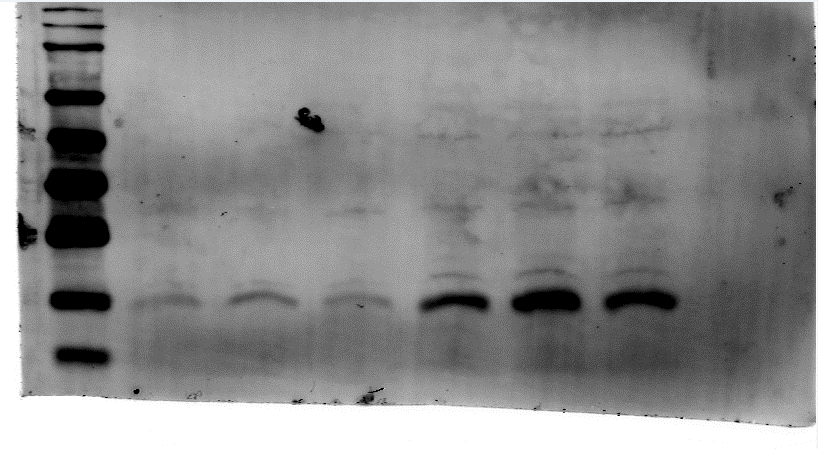

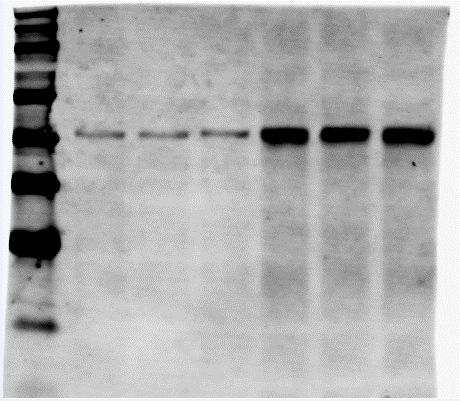


ccl-2

GAPDH


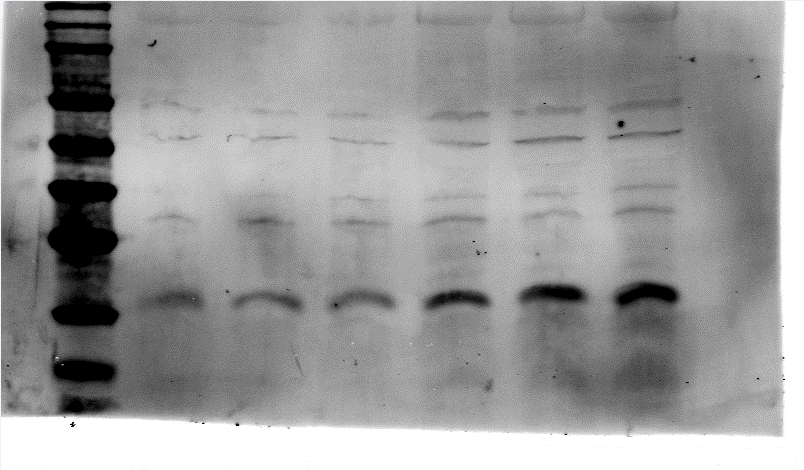


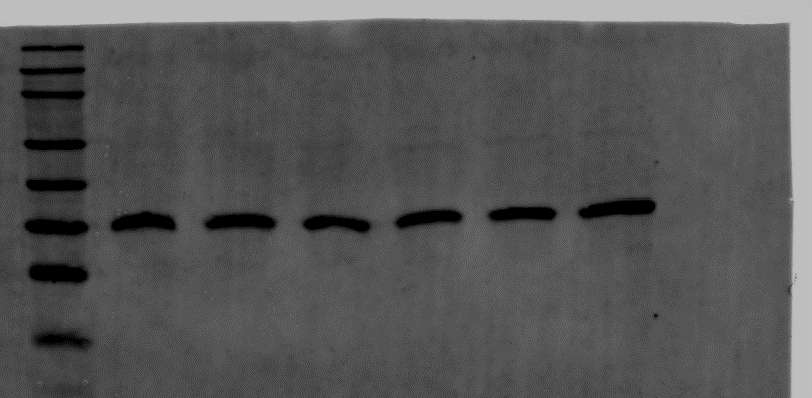


Figure3h

B7-1


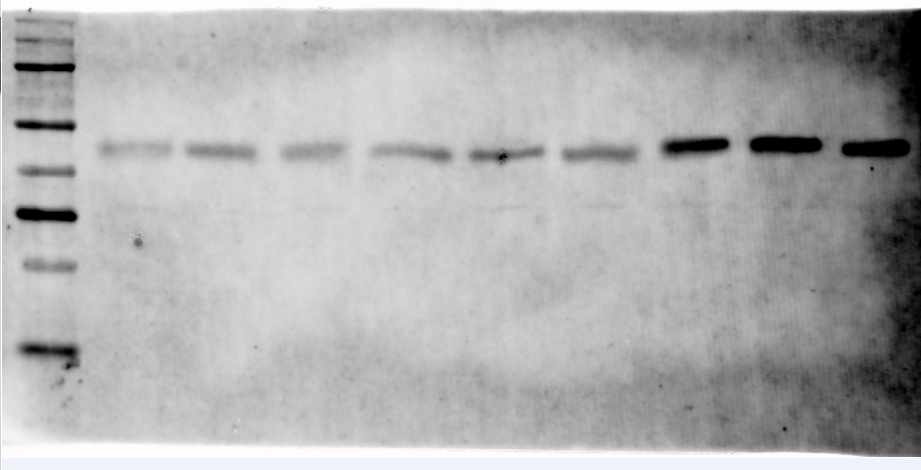

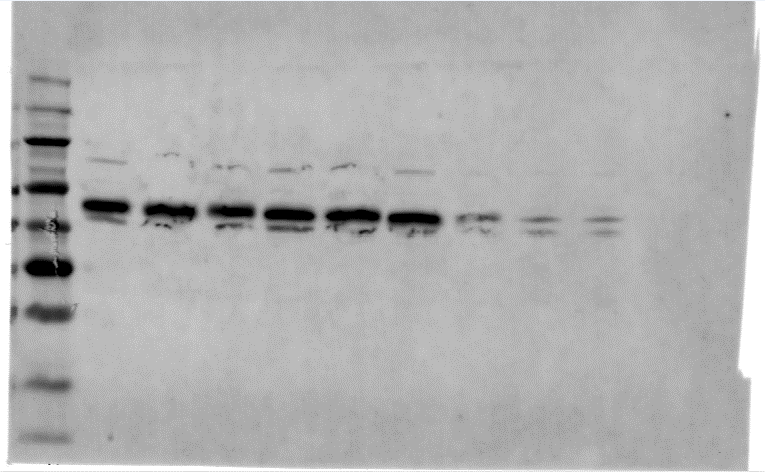


Podocin


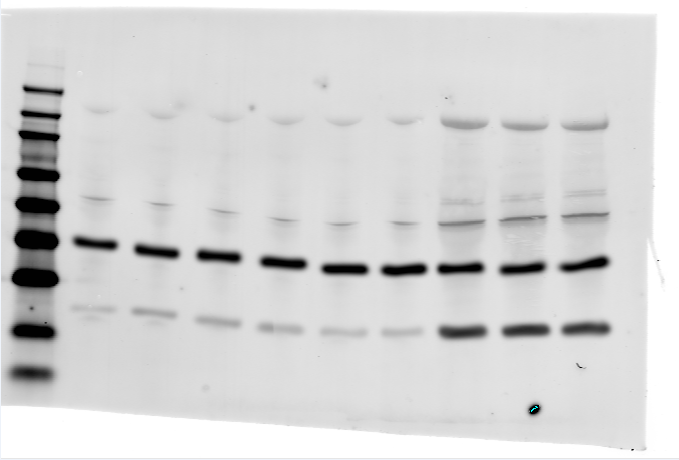


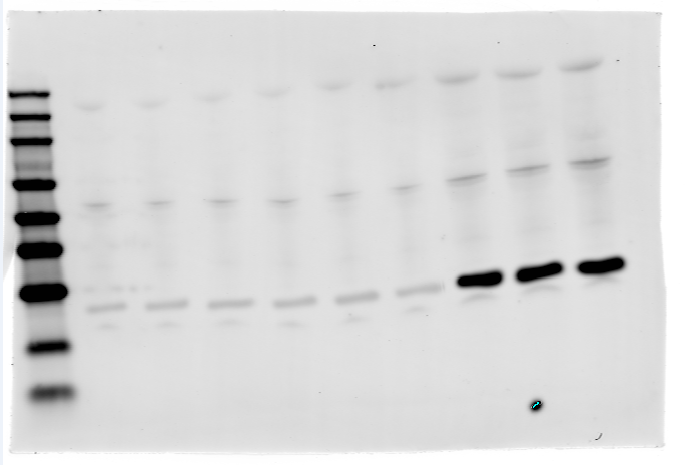


Claudin-1

GAPDH

ccl-2

IL-17


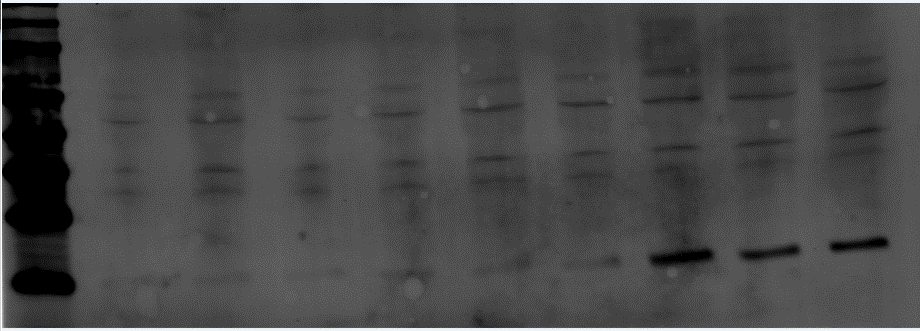


Figure4h

Claudin-1

Podocin


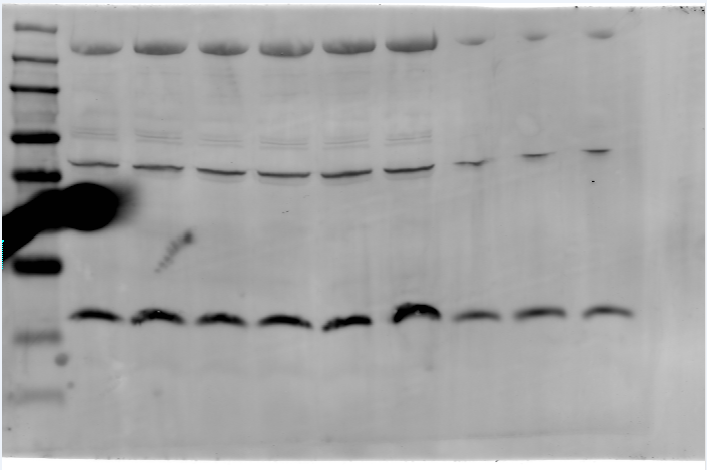

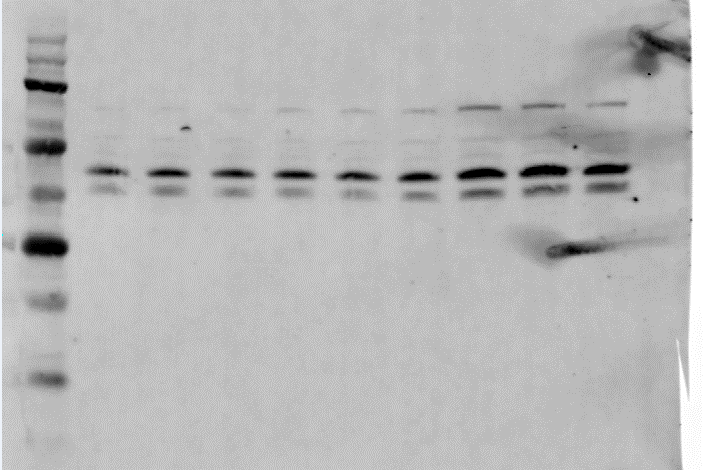

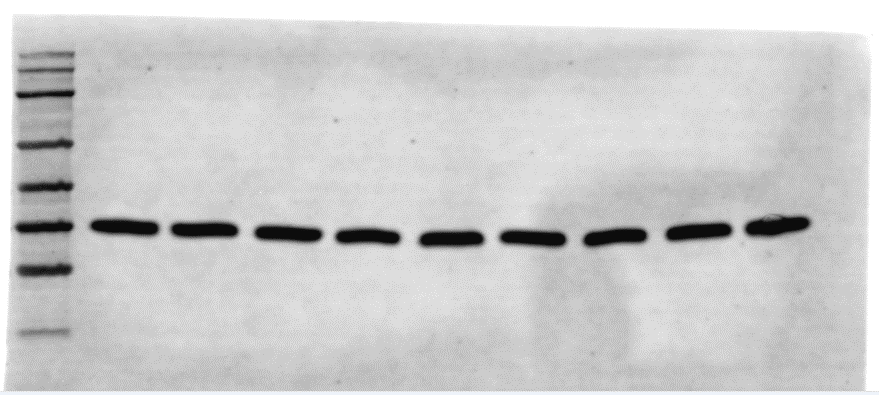


B7-1


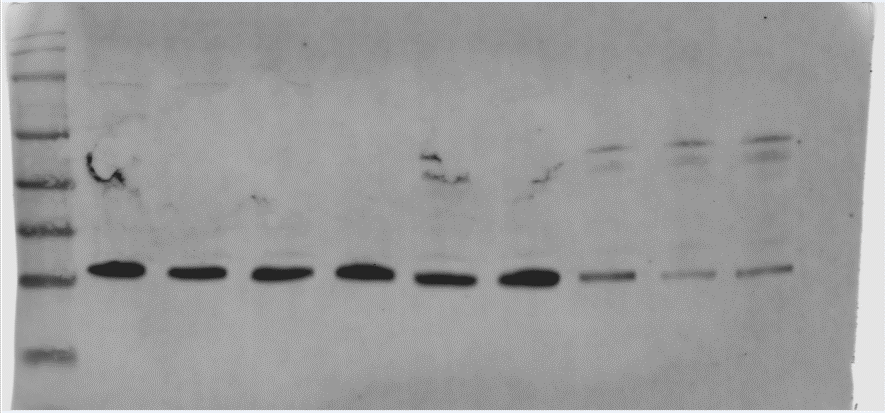

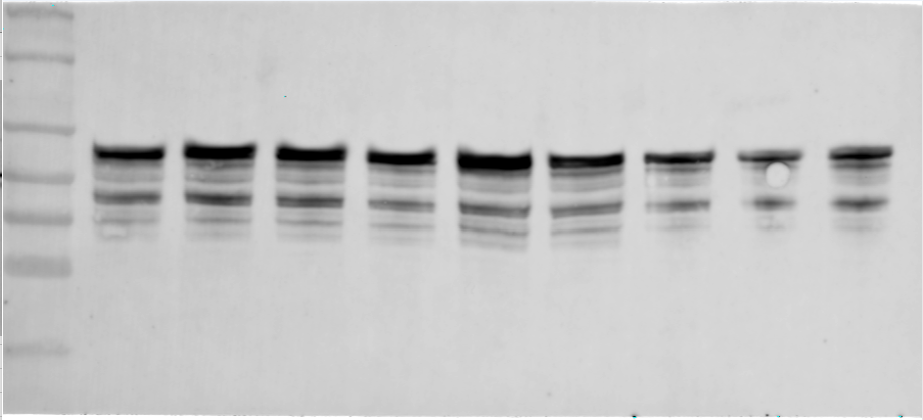


ccl-2

IL-17


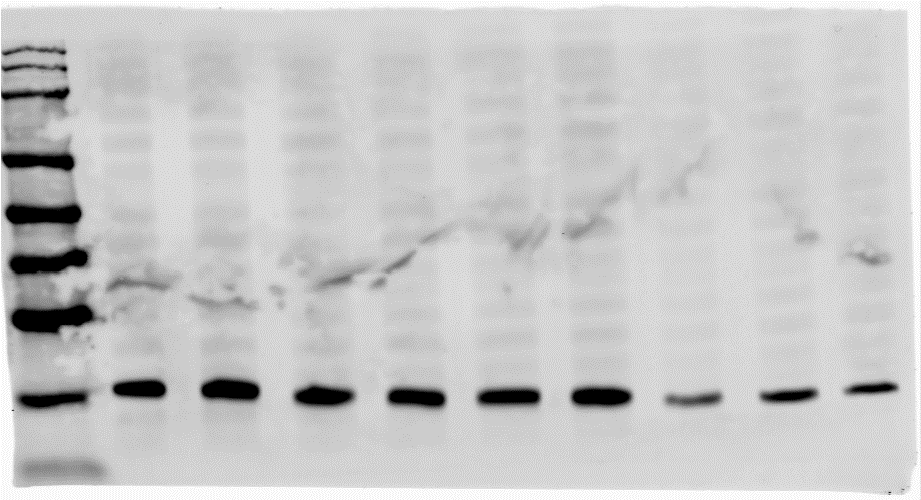


GAPDH


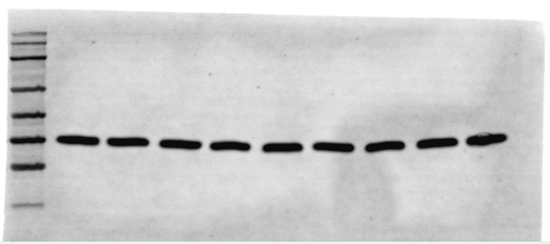


Figure5q

B7-1


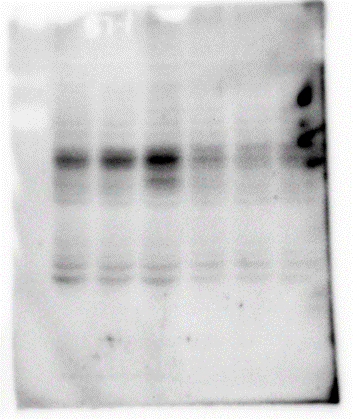


Podocin


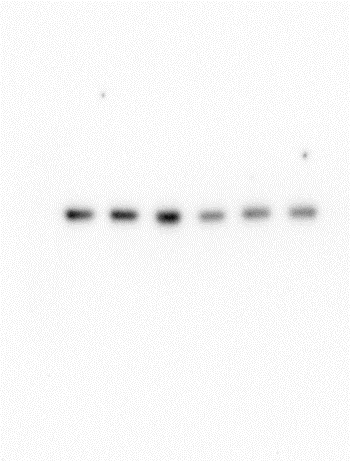

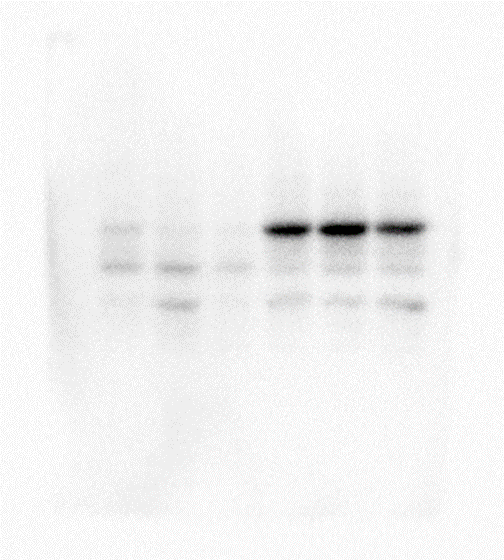


ccl-2

Claudin-1


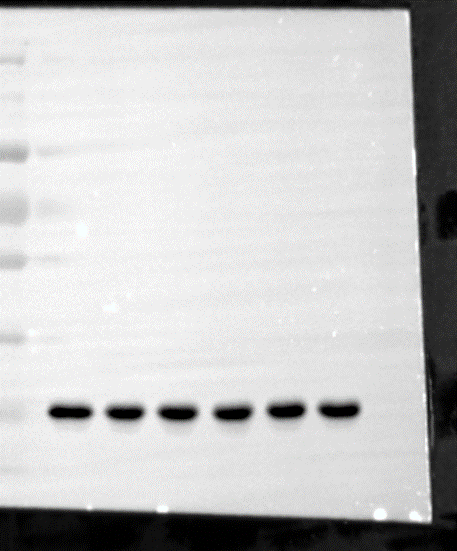

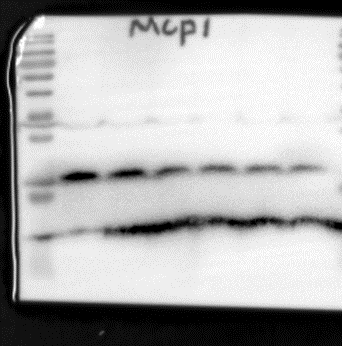

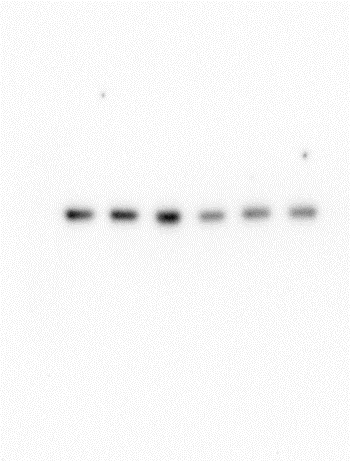


IL-17


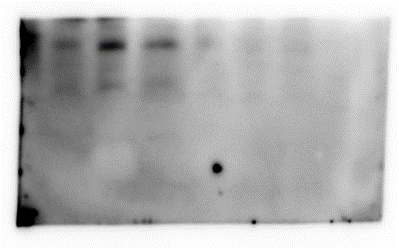


GAPDH

Figure6c

Podocin

1 2 3


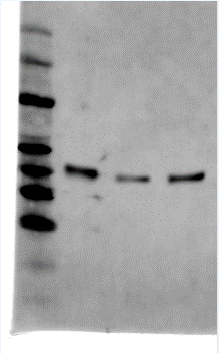

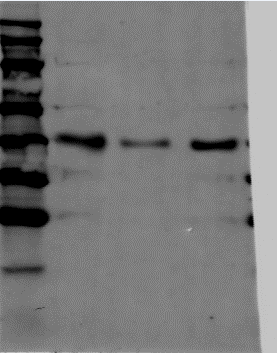

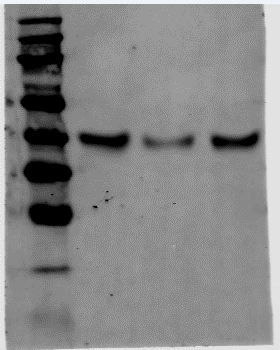

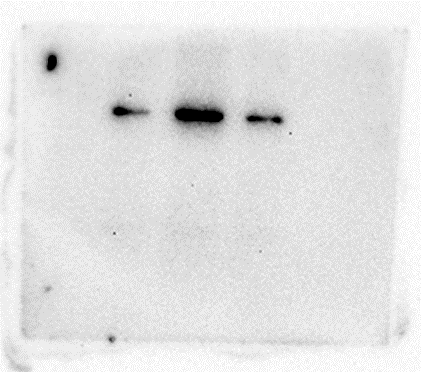


B7-1

1 2 3


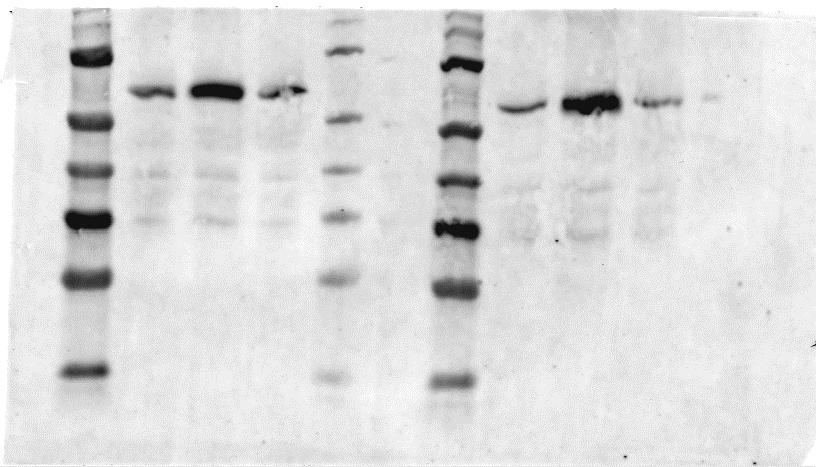

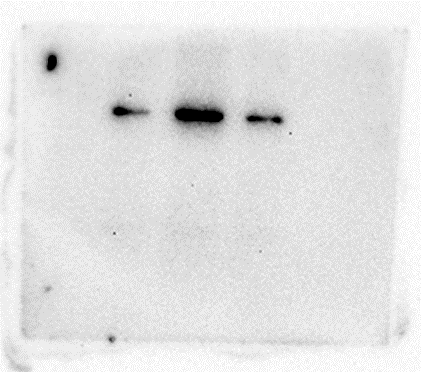


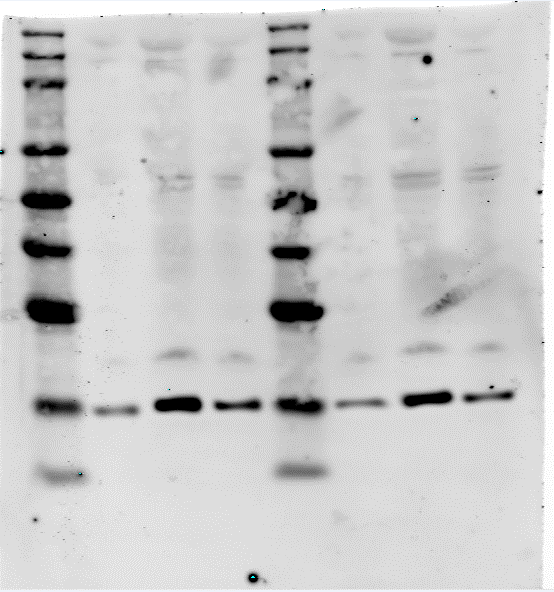
 1 2 3


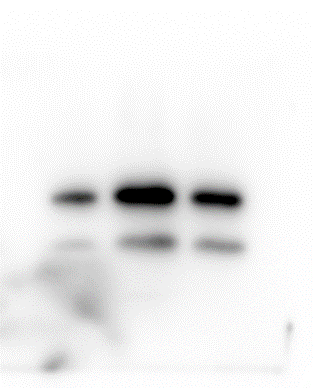


ccl-2


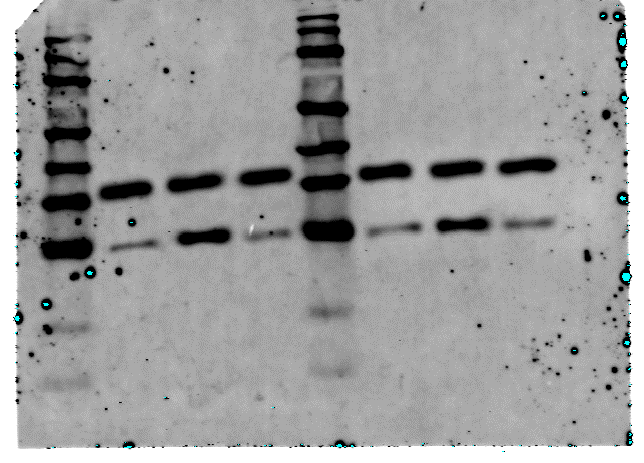
 1 2 3


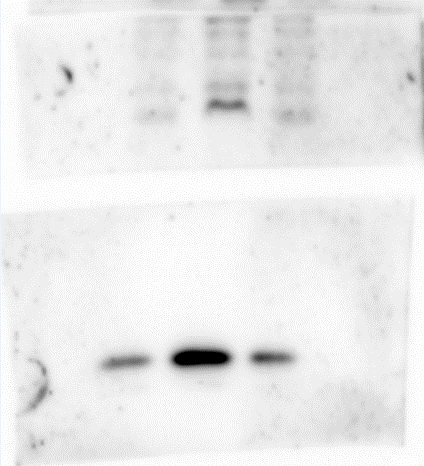


GAPDH

Claudin-1

Claudin-1

GAPDH

3


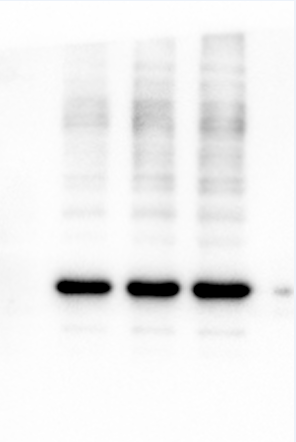


IL-17

1 2 3


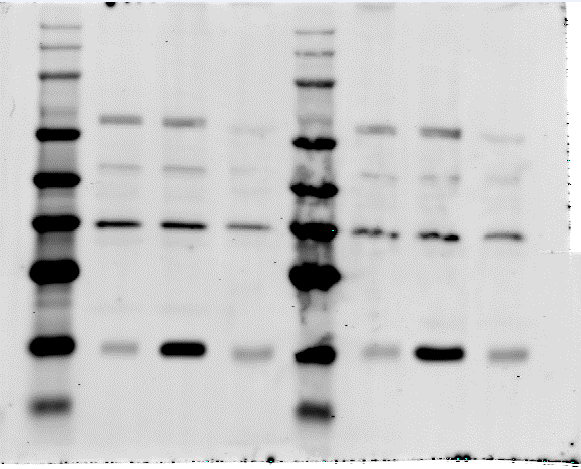

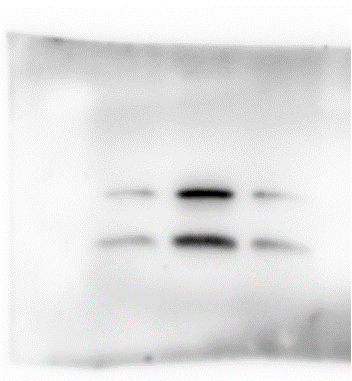


Figure6h

1 2 3


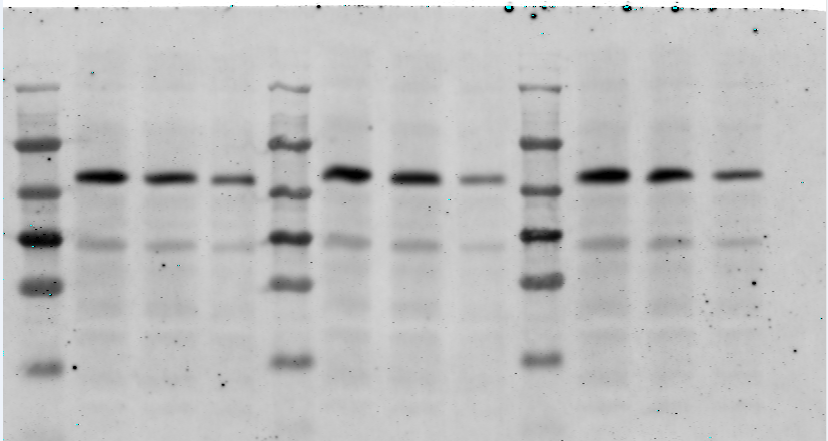


Podocin

1 2 3


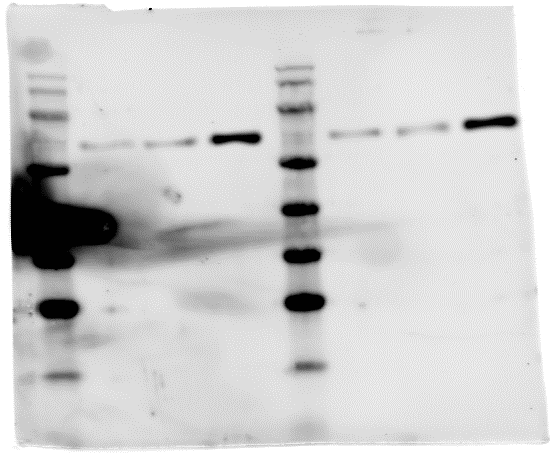

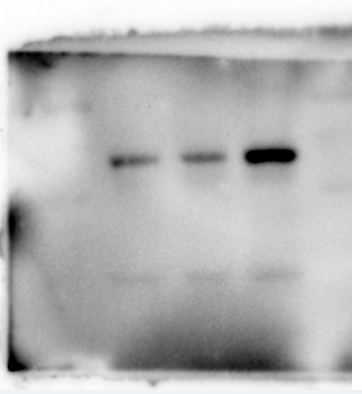


B7-1

Ccl2

1 2 3


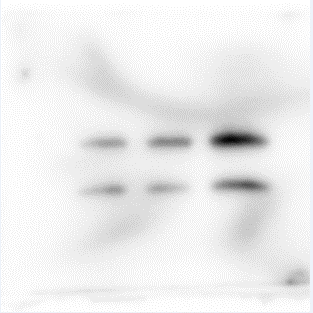

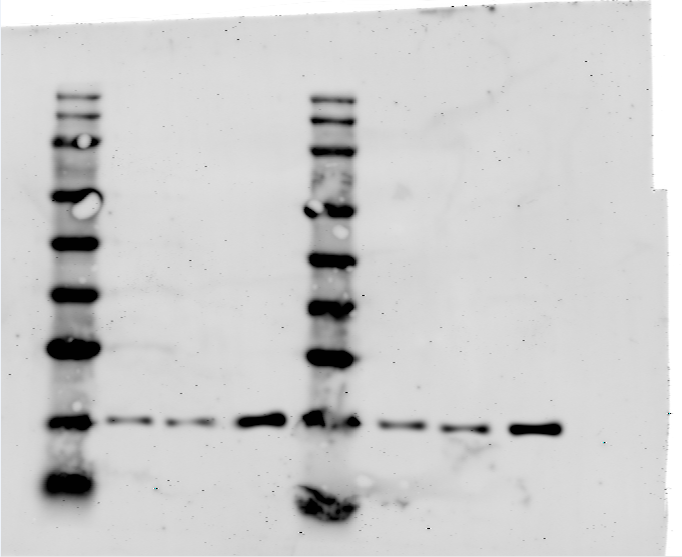


1 2 3


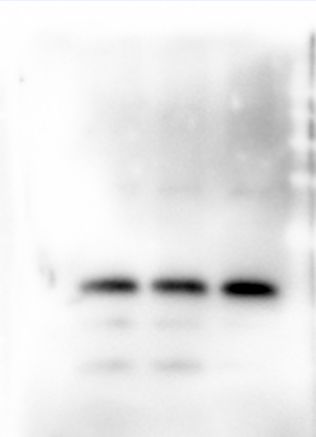

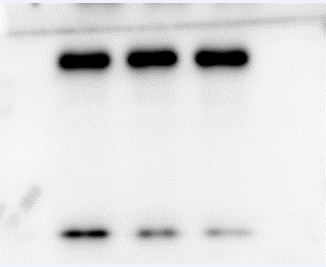

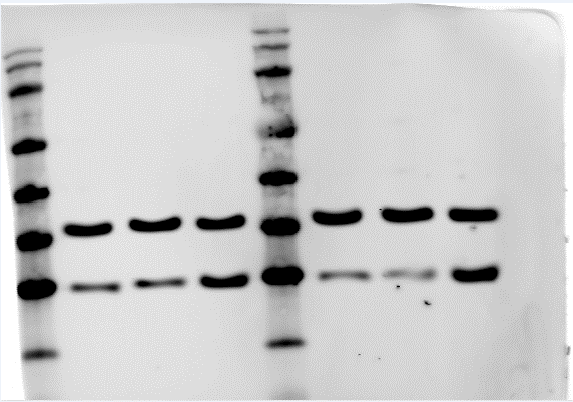


GAPDH

Claudin-1

Claudin-1

3

GAPDH

IL-17

1 2 3


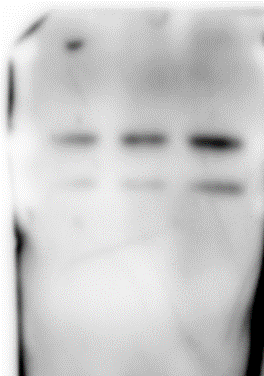

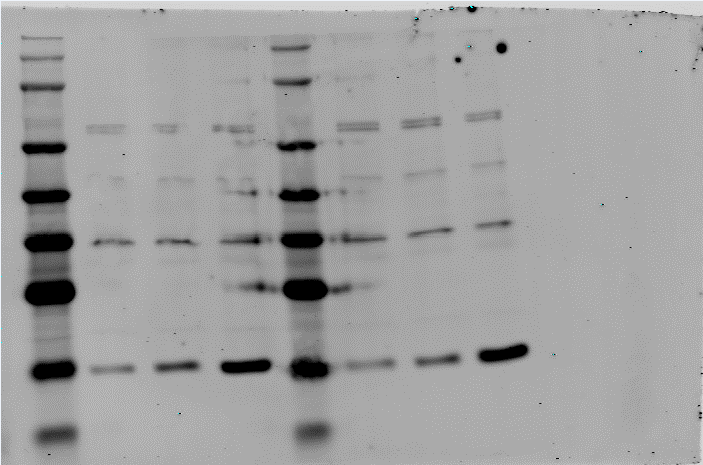


Figure6l

1 2 3

Podocin


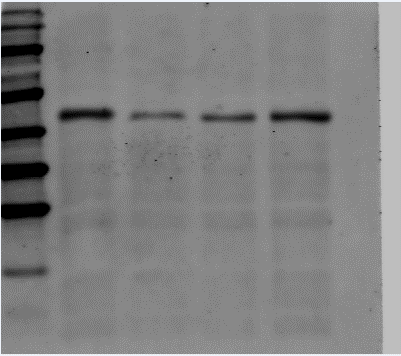

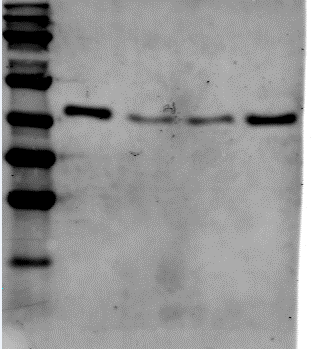

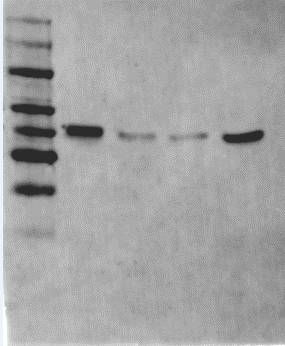


Claudin-1


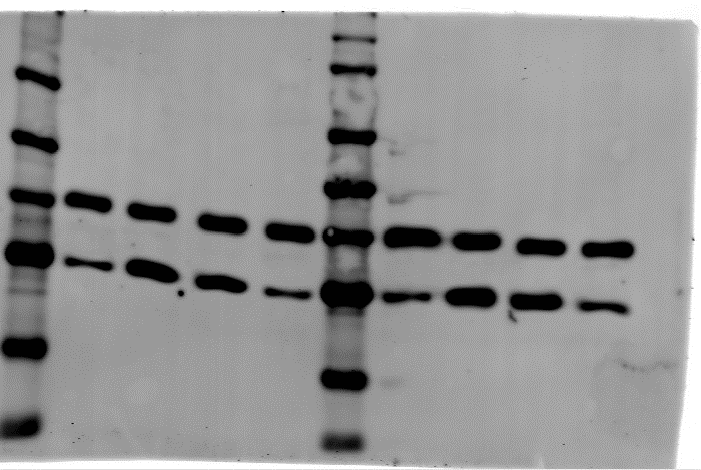
1 2 3


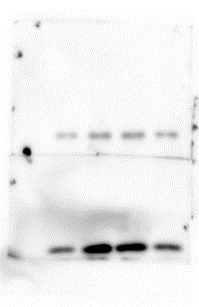


GAPDH


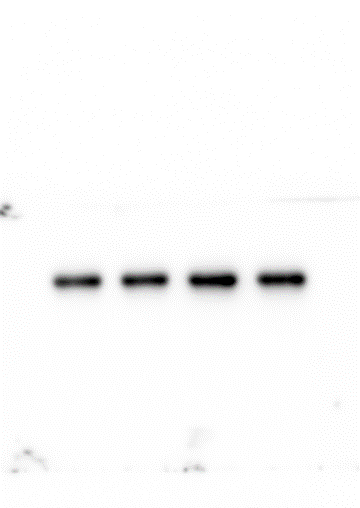


GAPDH

1 2 3

B7-1


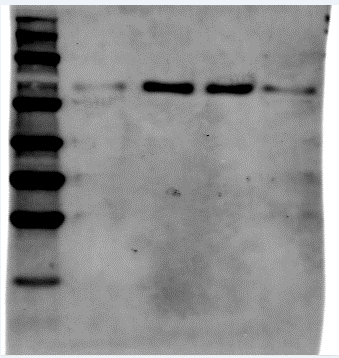

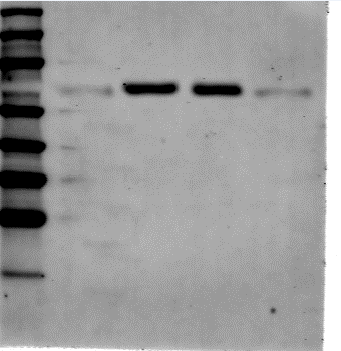

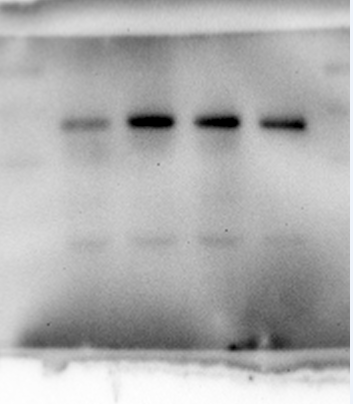


Ccl-2

1 2 3


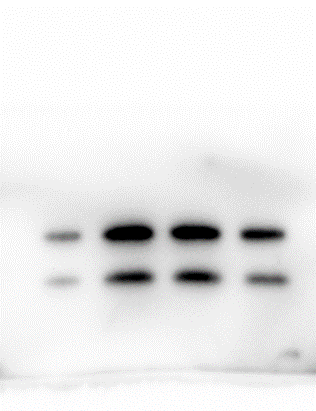

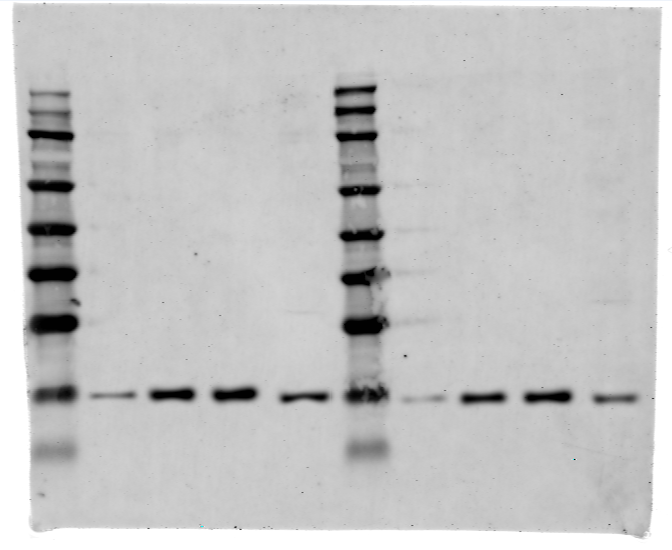


IL-17

1 2 3


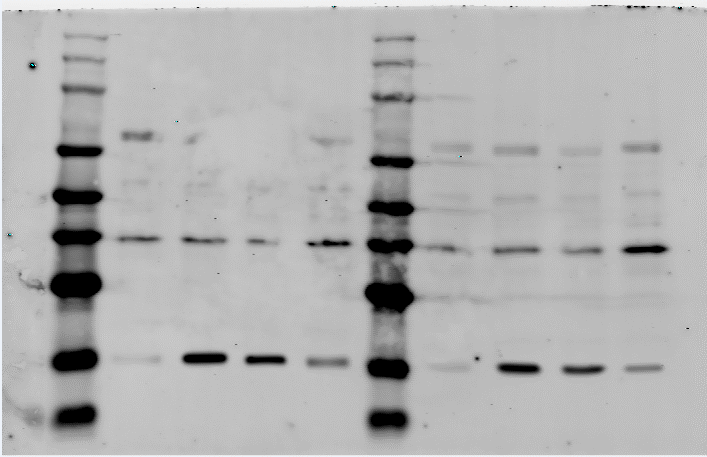

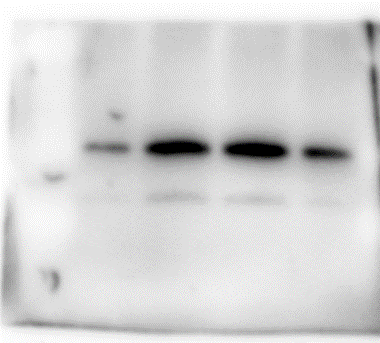


Figure7h

GSK-3β

1 2


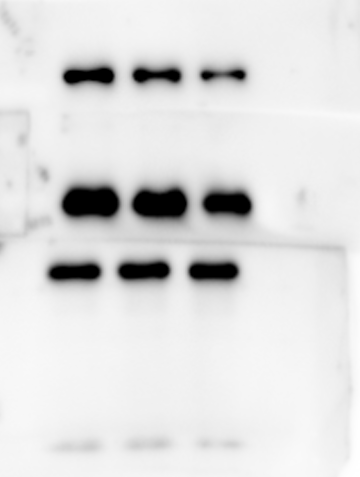

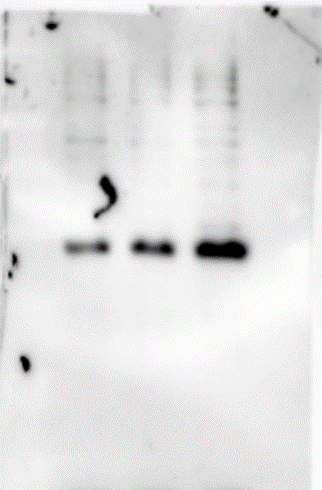

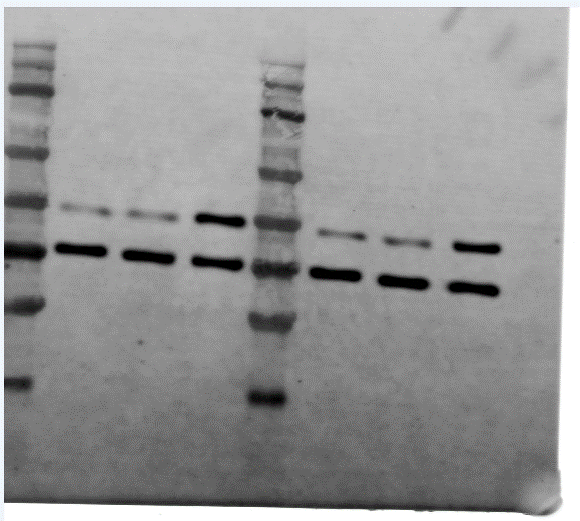


GSK-3β

GAPDH

GAPDH

Figure7i

1 2 3


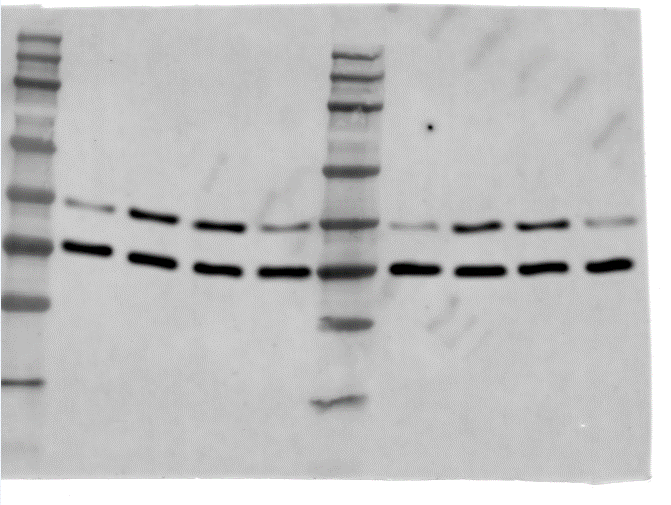

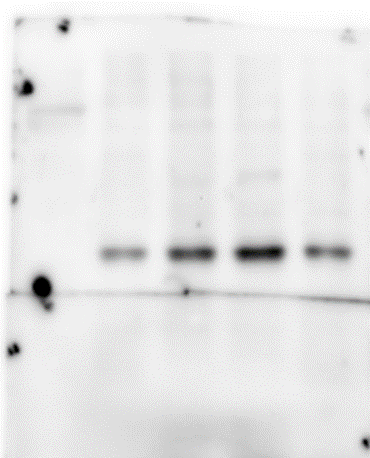




GSK-3β

GAPDH

GAPDH

GSK-3β

Figure7j

GAPDH

GSK-3β


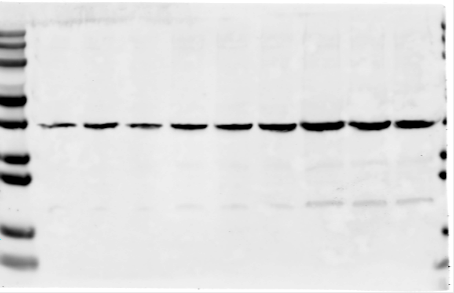

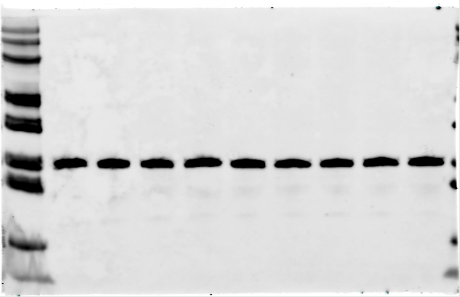


Figure7k


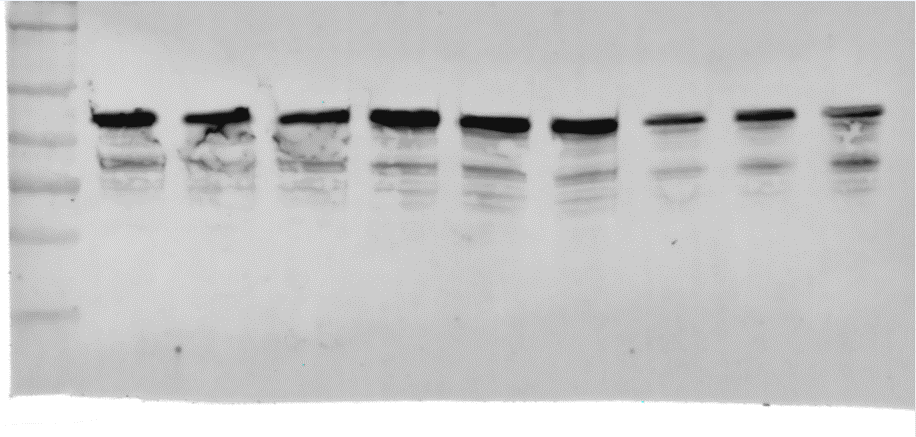


GSK-3β


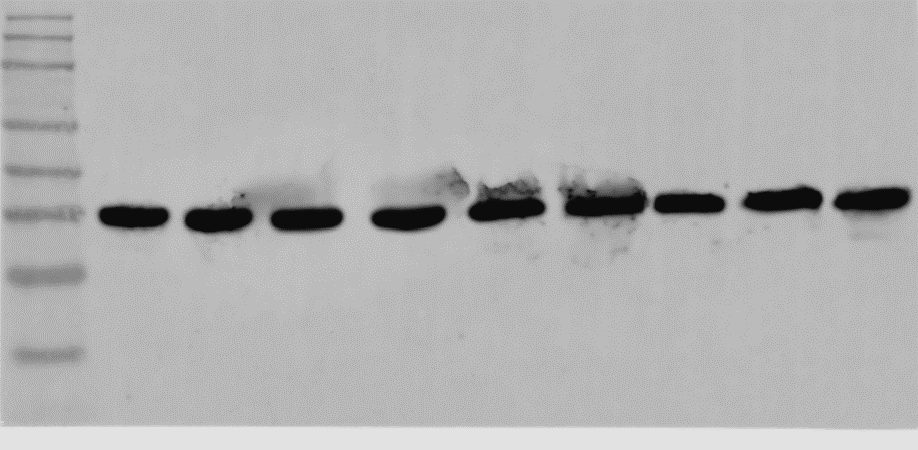


GAPDH


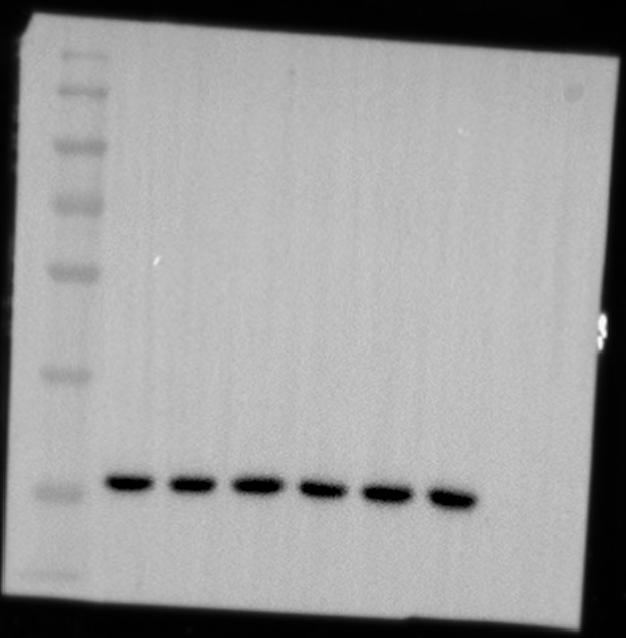
Figure8b


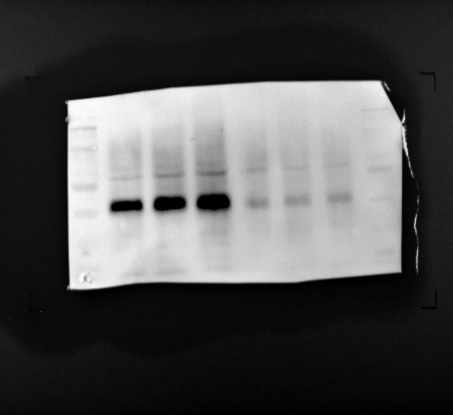


GAPDH

GSK-3β


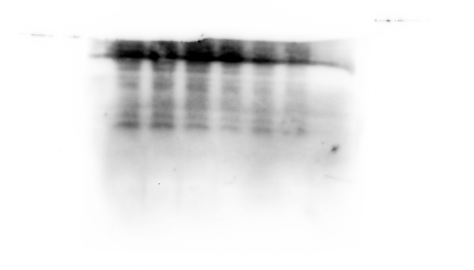
Figure8g

B7-1

Ccl-2


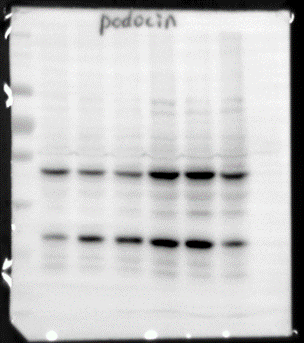

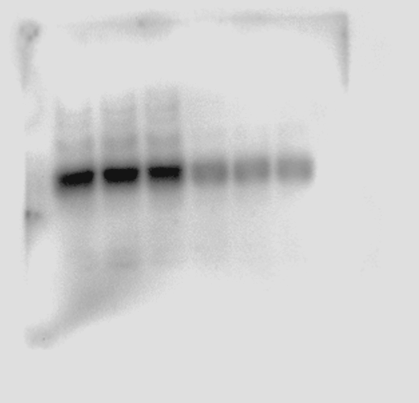


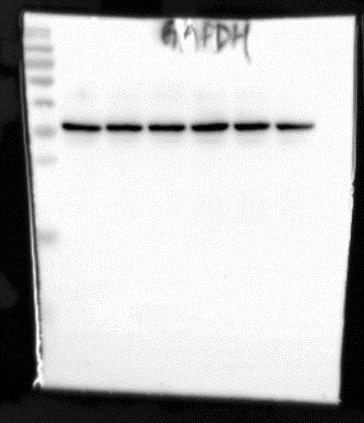


IL-17

Claudin-1


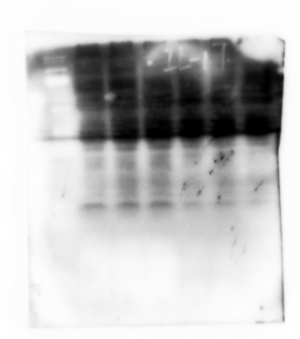

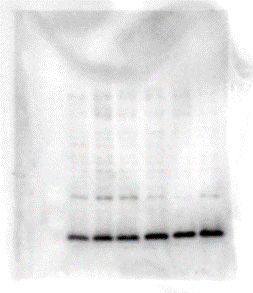

Supplement: Supplementary file 3 — Original Data File [file 41419_2023_5695_MOESM3_ESM.docx]
